# Glucose Signaling-Mediated Coordination of Cell Growth and Cell Cycle in *Saccharomyces Cerevisiae*

**DOI:** 10.3390/s100606195

**Published:** 2010-06-21

**Authors:** Stefano Busti, Paola Coccetti, Lilia Alberghina, Marco Vanoni

**Affiliations:** Dipartimento di Biotecnologie e Bioscienze, Università di Milano Bicocca, Piazza della Scienza, 2–20126 Milano, Italy; E-Mails: stefano.busti@unimib.it (S.B.); paola.coccetti@unimib.it (P.C.); lilia.alberghina@unimib.it (L.A.)

**Keywords:** glucose sensing, yeast, cell cycle, cAMP, PKA, Rgt2/Snf3, Snf1

## Abstract

Besides being the favorite carbon and energy source for the budding yeast *Sacchromyces cerevisiae*, glucose can act as a signaling molecule to regulate multiple aspects of yeast physiology. Yeast cells have evolved several mechanisms for monitoring the level of glucose in their habitat and respond quickly to frequent changes in the sugar availability in the environment: the cAMP/PKA pathways (with its two branches comprising Ras and the Gpr1/Gpa2 module), the Rgt2/Snf3-Rgt1 pathway and the main repression pathway involving the kinase Snf1. The cAMP/PKA pathway plays the prominent role in responding to changes in glucose availability and initiating the signaling processes that promote cell growth and division. Snf1 (the yeast homologous to mammalian AMP-activated protein kinase) is primarily required for the adaptation of yeast cell to glucose limitation and for growth on alternative carbon source, but it is also involved in the cellular response to various environmental stresses. The Rgt2/Snf3-Rgt1 pathway regulates the expression of genes required for glucose uptake. Many interconnections exist between the diverse glucose sensing systems, which enables yeast cells to fine tune cell growth, cell cycle and their coordination in response to nutritional changes.

## 1. Introduction

**Figure 1.**
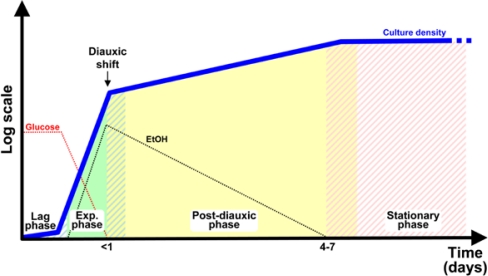
Growth phases of *S. cerevisiae* cultivated in rich medium supplemented with glucose. When quiescent, stationary phase cells are inoculated in fresh medium, they exhibit an initial lag phase of variable length. During the subsequent exponential phase cells proliferate rapidly by fermenting glucose to ethanol. When glucose becomes limiting, cells transiently arrest growth to adjust their metabolism from fermentative to the respiratory mode (diauxic shift): after the switch to respiration, cells restart growing at a reduced rate by slowly consuming the ethanol accumulated in the medium. When ethanol is also exhausted, cells cease dividing and enter into a quiescent state known as stationary phase that becomes deeper and deeper as cells spend more time in this state. Solid colors indicate steady states, diagonal stripes transient states.

The budding yeast *Saccharomyces cerevisiae* is the first eukaryote whose genome was completely sequenced [1] and its ease of manipulation and the wide array of molecular and post-genomic techniques available make it a preferred model organism for genetic, biochemical and, more recently, systems biology studies [2–4]. *S. cerevisiae* can be grown both in batch and continuous cultures under a variety of conditions that allow modulation of its physiological response. In any given growth medium supporting cellular proliferation, a time window can be defined in batch cultures in which macromolecular syntheses and cell division are coordinated, so that any given intracellular parameter, such as protein or DNA distribution in the population, is constant. This so-called “balanced exponential growth” (solid green area in Figure 1), is usually preceded by a lag phase (white and green stripes in Figure 1) and followed by a transient phase leading to stationary phase. Upon nutrient exhaustion yeast cells enter into a non-proliferating, quiescent state (white and red stripes in Figure 1), characterized by strongly diminished transcriptional and protein synthesis rate, severely reduced expression of genes encoding ribosomal proteins and induced transcription of stress responsive genes, accumulation of storage carbohydrates, thickened cell wall, enhanced stress resistance, chromosomes condensation and autophagy (the process of engulfment of the cytoplasm into lipid vesicles which are delivered to the vacuole for degradation) [5].

When cells are grown on glucose and no other nutrient is limiting, then a second phase of growth takes place where yeast cells use the ethanol they produced during the first phase of growth. This pattern of growth (called post-diauxic growth, solid yellow area in Figure 1) takes place because despite the presence of oxygen, yeast cells metabolize glucose by alcoholic fermentation, rather than fully oxidize glucose to water and carbon dioxide via the TCA cycle and turn to fermentation only when oxygen becomes limiting, as most cells do. Although energetically less efficient than respiration, fermentation can proceed at much faster rates, allowing budding yeast to aggressively utilize glucose at the expenses of its energetically efficient but slower competitors: the rapid depletion of the sugar and the accumulation of large amounts of ethanol produced during fermentation (which is toxic for most competing microorganisms) enable yeast cells to successfully compete for survival.

To be effective, the above-described strategy requires accurate monitoring of extracellular conditions and a fast and coordinate way to regulate gene expression so to optimize glucose utilization and achieve the highest possible growth rate by fine tuning cell growth (*i.e.*, the increase in cell mass at the single cell level) and cell cycle, (*i.e.*, those discontinuous event that take place only once within each cell division and that include DNA replication, chromosome segration and cell division itself).

Since glucose is its preferred carbon source and it is central to its ability to survive in the wild, *S. cerevisiae* has evolved a sophisticated system for sensing of glucose (both outside and inside the cell) and its uptake. Here we will review the major properties of glucose sensing and transport systems and will discuss some novel findings that highlight a major and previous unrecognized role of glucose sensing in controlling cell growth, cell cycle and their coordination.

## 2. Glucose Transport in *S. Cerevisiae* Relies on a Multi-Component Uptake System

Glucose import into the yeast cell occurs via facilitated diffusion through a group of membrane-spanning proteins, termed hexose transporters (encoded by *HXTs*) [6,7]. *S. cerevisiae* possesses at least 20 glucose transporter (*HXT1* to *HXT17*, *GAL2*, *SNF3* and *RGT2*), whose sequence alignment reveals a high degree of conservation throughout the regions comprising the 12 predicted trans-membrane segments. The amino- and carboxyl-terminal tails, both localized on the cytosolic face of the plasma membrane, differ considerably in length and aminoacidic composition.

None of the transporters is individually essential for viability or growth on glucose [8]. The transporters encoded by *HXT1* to *HXT7* seem to the be most metabolically relevant, since a strain lacking these seven genes (often designed as “*hxt*-null” mutant, [8,9] is unable to grow on glucose as a sole carbon source and has no detectable glycolytic flux, suggesting that the remaining carriers (*HXT8-17*] do not contribute significantly to glucose import, perhaps as a consequence of their reduced expression level (at least under the most common growth conditions) [7]. However, when individually overexpressed in a *hxt*-null strain, all *HXTs* gene products support growth on glucose, although to a variable extent [10]; the only exceptions are *HXT12* (a possible pseudogene), and *SNF3* and *RGT2*, which act as glucose sensors, but lost ability to transport sugar [10,11]. The presence in yeast of a multi-factorial glucose uptake system may reflect the need of this microorganism to deal with the extremely broad range of sugar concentrations occurring in its natural habitat; in fact, yeast cells can efficiently metabolize glucose over a broad concentration range, from a few micromolar to a few molar. The major hexose transporters in *S. cerevisiae* (encoded by the *HXT(1-7)* genes) cover the whole affinity range for glucose from 1 to 100 mM (*K_m_*) and have been classified as high affinity (*K_m_* ≈ 1 mM: Hxt7, Hxt6 and Gal2), medium affinity (*K_m_* ≈ 5–10 mM: Hxt2 and Hxt4) and low-affinity (*K_m_* ≈ 50–100 mM: Hxt1 and Hxt3) glucose carriers [12]. Hxt2 is quite atypical, since it exhibits biphasic uptake kinetics with a low- and high-affinity component on low glucose and an intermediate affinity on high glucose concentration [8,12].

Since the diverse carriers exhibit different kinetic properties, each of them appears particularly suited for a specific growth condition: for instance, Hxt1, a low affinity, high capacity transporter, is most useful when glucose is abundant, whereas Hxt6 and Hxt7, two high affinity carriers, are necessary when the sugar is scarce [8,9].

Yeast cells express only the glucose transporters most appropriate for the amount of sugar available at any moment in the environment. This pattern is due to the combined action of different regulatory mechanisms, including transcriptional regulation of the major *HXT* genes in response to glucose [11,13–15] and inactivation of Hxt proteins under appropriate conditions [16–19]. A minor contribute may also arise from the modulation of the affinity for the sugar of certain transporters (*i.e.*, *HXT2*) [6,9,12]. At least five types of transcriptional regulation by different level of glucose have been described for the major *HXT* genes: *(i)* induction by high concentrations of glucose (*HXT1*) [13,20,21]; *(ii)* induction only by low levels of glucose (*HXT2* and *HXT4*) [21,22]; *(iii)* induction independent by glucose concentration (*HXT3*) [13,20,21]; *(iv)* repression by high level of glucose (*HXT6* an *HXT7*) [20,21,23–26]; *(v)* regulation independent by glucose concentration (*HXT5*) [20,21,27–30].

The transcriptional regulation of *HXT* genes is the result of a complex interplay between at least two different pathways which constantly monitor the levels of glucose. Glucose induction of *HXT* genes is dependent on the Snf3/Rgt2-Rgt1 pathway [11,14,22,31–33], for which few other genes have been validated as targets [21,34]. The glucose repression circuit that operates through the Snf1 protein kinase and the Mig1 transcriptional repressor prevents the expression of the high/intermediate affinity hexose carriers (encoded by *HXT2*, *HXT4*, *HXT6* and *HXT7)* when the sugar levels are high [15,22]. The function of Rgt1, a central player in the Snf3/Rgt2 circuit, can also be modulated through phosphorylation by a third glucose-sensing pathway, the cAMP/PKA pathway [33].

Other mechanisms appear to contribute to regulation of *HXT* expression: for example, roles for the HOG pathway [35,36] and the TOR network [37] in the transcriptional regulation of *HXT1* have been proposed. Glucose phosphorylating enzymes (in particular Hxk2) also appear to influence the expression pattern of the *HXT* genes [7,13,38–41].

## 3. Sensing Extracellular Glucose

### 3.1. Glucose Induction Signal: the Snf3-Rgt2 Signaling Transduction Pathway

#### 3.1.1. The Snf3 and Rgt2 Sensors

**Figure 2.**
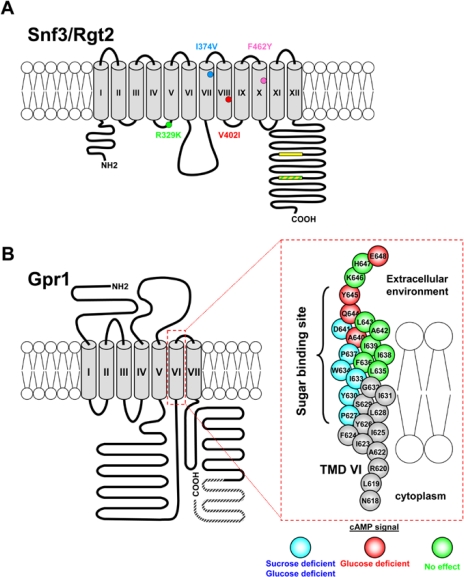
Structure of the Gpr1 Glucose Receptor and of the Snf3/Rgt2 Glucose Sensors. (a) The two-dimensional transmembrane topology of the Rgt2p and Snf3p glucose sensors, based on the model of the mammalian Glut1 glucose transporters. The predicted transmembrane domains are numbered I to XII. Aminoacidic substitutions which alter glucose signaling are depicted as coloured dots (see main text for details). Yellow box indicates a repeated sequence of 25 amino acids in the carboxyl-terminal tail of both Rgt2p and Snf3. A second copy (yellow/green shaded box) is also present in the Snf3 carboxy-terminus. (b) The putative structure of Gpr1 model is based on the similarity with GPCRs found in higher eukaryotes [89]. The predicted transmembrane domains are numbered I to VII. The C-terminal 99-amino-acid sequence interacting with Gpa2 is highlighted. The magnification of the transmembrane domain VI shows the residues (likely located adjacent to the putative sugar binding site) whose replacement with cystein affects the glucose induced cAMP signaling or both the glucose- and saccharose-induced cAMP signaling. Images adapted from references 14, 87 and 90.

Despite their high similarity to the Hxt glucose transporters, Snf3 and Rgt2 seem to have lost the ability to import sugars inside the cell [10,11] and function instead as receptors that monitor extracellular glucose: in particular, Rgt2 seems to be a low affinity receptor required for maximal induction of *HXT1* (low affinity carrier) by high glucose, while Snf3 (Figure 2A) is a high affinity sensor needed for the transcription of *HXT2* and *HXT4* (moderately affinity carriers) genes in response to low levels of glucose [11,22].

Distinctive features of both Snf3 and Rgt2 are their long cytoplasmic C-terminal tails (∼200 aa) that play an important role in glucose signaling [11,24,42]. The C-terminal extensions of the two receptors are quite dissimilar, except for a brief sequence motif (∼25aa, yellow boxes in Figure 2A) that occurs twice in the Snf3 tail and once in Rgt2 and that is apparently required for the signaling function: in fact, deletion of this conserved motif, as well as of the whole C-terminal domain, impairs the ability of Snf3 and Rgt2 to induce the expression of *HXT* genes in response to glucose. The C-terminal extensions are sufficient for glucose signaling, since attaching them to a Hxt transporter confers on it glucose signaling ability [11]; furthermore, the expression of isolated tail domains (fused to a membrane-targeting sequence or as soluble proteins) leads to a constitutive glucose signal [24,42,44]. However, the tails are not strictly necessary for signaling, since a tail-less version of Rgt2 is functional when overexpressed [44].

**Figure 3.**
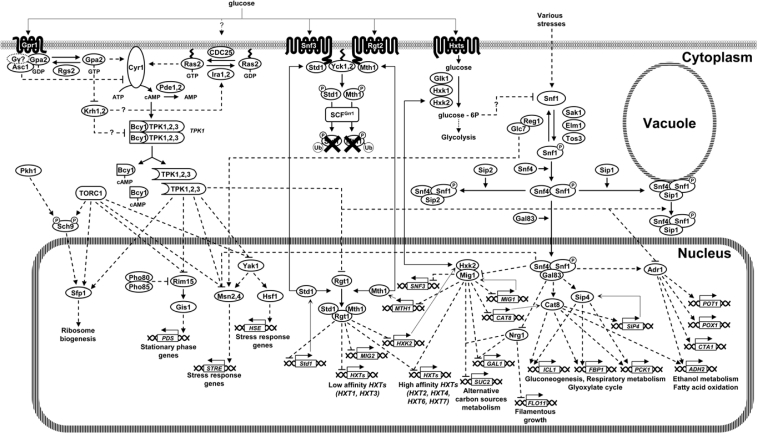
A simplified view of the glucose sensing mechanisms in *S. cerevisiae*. A schematic view of the three glucose sensing pathways in yeast. The major interconnections with the TOR pathway (only partially shown) are indicated. See text for details.

It is generally accepted that glucose signaling by Snf3 and Rgt2 is a receptor-mediated process similar to hormone signaling in mammalian cells [11,14,15,], the glucose signal being generated by the transmembrane domain of the glucose receptors upon binding of the sugar, while the C-terminal tails may enhance signaling by facilitating the recruitment of the Mth1 and Std1 co-repressors (see Section 3.1.2. and Figure 3) to the plasma membrane [44]. A dominant mutation in the Snf3 and Rgt2 sensors—consisting in the replacement by a lysine of a conserved arginine localized in the cytoplasmic loop preceding the 5th transmembrane helix (R229K (R231K in Rgt2; green dot in Figure 2A)—leads to the constitutive expression of *HXT* genes even in complete absence of glucose, possibly locking the receptors in a conformation corresponding to that of the glucose-bound form [14]. Snf3 ligand specificity is not limited to glucose, since it senses fructose and mannose, as well as different glucose analogues. By site-specific mutagenesis of the structural gene, roles of specific Snf3 residues in sensing specificity have been established [46]. A V402I substitution (red dot in Figure 2A; V404I in Rgt2) virtually abolishes glucose signaling: since the corresponding mutation in Hxt1 (F371) blocks glucose transport this residue may be part of the glucose binding domain [44]. The I374V substitution (light blue dot in Figure 2A) partially abolishes sensing of fructose and mannose by Snf3, whereas the F462Y mutation (purple dot in Figure 2A) abolishes sensing of fructose. Neither of these amino acid changes affects glucose signaling [46].

#### 3.1.2. Downstream Elements of the Snf3-Rgt2 Signaling Transduction Pathway

The central players downstream of Snf3 and Rgt2 are Rgt1, a transcriptional repressor which negatively regulates the expression of *HXT* genes and the *SCF^Grr1^*ubiquitin ligase complex, that inhibits the activity of the Rgt1 repressor as described below.

Rgt1 contains a C_6_-(Cys_6_ Zn_2_) ‘zinc cluster’ DNA-binding domain in its N-terminus [47]. In the absence of glucose [47–50] Rgt1 binds synergistically to multiple sites found in the upstream regions of most *HXT* genes blocking their transcription by recruiting the general repressors Ssn6 and Tup1 [31,51]. Rgt1 transcription repressing activity requires Mth1 and Std1 [25,50,52–54].

The two co-repressors Mth1 and Std1 bind to a common site on Rgt1: Mth1 serves primarily to maintain repression of *HXT* in absence of glucose (*i.e.*, during growth on non-fementable carbon sources), whereas Std1 may play a role in the establishment of repression when the available glucose is exhausted [32,34,55]; see also 5.1. While Mth1 and Std1 bind to Rgt1 inside the nucleus [54] and to the glucose sensors at the cell surface [52,53], so far there is no evidence that their subcellular localization is regulated [15], although evidence that Snf1 kinase might favor their nuclear retention has been reported [56].

When glucose becomes available, it binds to the Snf3/Rgt2 sensors on the plasma membrane [11,14] leading to phosphorylation of Mth1 and Std1, priming them for SCF^Grr1^-mediated ubiquitination that targets the co-repressors to the 26S proteasome [32,33,44,50,57]. Phosphorylation of the corepressors requires activation of Yck1 (and its paralogue Yck2) a membrane-anchored type I casein kinase involved in many cellular processes [44]. The necessity of a direct coupling between the glucose sensors and Yck1/2 for the transmission of the glucose signal from the plasma membrane to the nucleus has recently been challenged, suggesting the involvement of a yet unidentified signaling component in the process [58]. Degradation of Mth1 and Std1 [32,50] exposes Rgt1 to phosphorylation by PKA allowing an intramolecular interaction between the central region of Rgt1 and its zinc-finger domain, thus inhibiting DNA binding of Rgt1 that is forced to leave the *HXT* promoters [32,33,44,47,49–51,54].

Multiple evidences support the notion that PKA contributes to glucose induction of *HXT* gene expression by catalyzing phosphorylation of Rgt1: i) PKA phosphorylates Rgt1 *in vitro*; ii) glucose fails to induce *HXT* genes expression in yeast cells with reduced PKA activity, whereas the transcription of *HXTs* is constitutive in strains with an hyperactive cAMP/PKA pathway; iii) several serine residues in the N-terminus of Rgt1, which are likely to be phosphorylated by PKA, are essential both for the intramolecular reaction of the repressor and for its release from the *HXT* promoter in response to glucose [33].

Therefore, two distinct glucose-induced events must occur for the removal of the Rgt1 repressor from the *HXT* promoters to take place: Mth1 and Std1 must be degraded via the Snf3-Rgt2 glucose-sensing pathway and Rgt1 must be phosphorylated via the cAMP/PKA glucose-sensing circuit. Yeast cells may take advantage of this strategy to induce different *HXT* genes in response to different levels of glucose. When glucose levels are low, Mth1 would be degraded, but Rgt1 would not be completely phosphorylated because PKA is not fully active under these conditions: this event might result only in induction of *HXT* genes encoding high affinity glucose transporters (e.g., *HXT2* and *HXT4*). When glucose levels are high, Mth1 would be degraded, and Rgt1 would be fully phosphorylated because PKA is fully active: this fact would drive to completion the intramolecular interaction of Rgt1 and result in full induction of the low affinity carriers (*i.e.*, *HXT1* and *HXT3*) [32,33].

### 3.2. The cAMP/PKA Pathway

In yeast, a major signaling pathway activated by glucose is the cAMP/protein kinase A pathway, which regulates many aspects of cellular physiology, including growth, proliferation, metabolism, stress resistance, aging, morphogenesis and development according to nutrients availability (Figure 3). Adenylate cyclase activity in *S. cerevisiae* is controlled by two distinct G-protein systems: the Ras pathway and the Gpr1-Gpa2 pathway [59–62]. Ras1 and Ras2 are two small monomeric GTP-binding proteins capable to switch between an active GTP-bound state and an inactive GDP-bound form. The Ras-GTP/Ras-GDP ratio is controlled by the balance between the activities of the guanine nucleotide exchange factors (GEFs), Cdc25 [63] and Sdc25 [64], which promote GTP loading on Ras, and the GTPase Activating Proteins (GAPs), Ira1 and Ira2, which stimulate GTP hydrolysis by enhancing the intrinsic Ras-GTPase activity [65,66]. When in their active conformation, Ras proteins stimulate cAMP production by direct binding to adenylate cyclase [67]. The level of cAMP in yeast cell is the result of the equilibrium between its synthesis, catalyzed by the adenylate cyclase enzyme, Cyr1 [68], and its degradation performed by the low- and high-affinity phosphodiesterases, encoded by *PDE1* and *PDE2*, respectively [69,70].

Ras proteins are required to maintain basal adenylate cyclase activity and are thus essential for cell viability. Besides intracellular acidification [71], glucose addition also causes a small but significant increase in the fraction of GTP-bound Ras [72–73] (see Section 3.2.2).

cAMP-dependent protein kinase (PKA) is a conserved serine/theonine kinase that exists in its inactive status as a heterotetrameric holoenzyme composed of two catalytic subunits (encoded in yeast by the three closely related genes: *TPK1*, *TPK2* and *TPK3*) and two regulatory subunits (encoded by *BCY1*) [74,75]. The three catalytic subunits of PKA are largely redundant, although several specific functions have also been described for each isoforms [76–80].

At least two different mechanisms regulate the subcellular localization of PKA: cAMP controls the localization of the Tpks catalytic subunits, whereas the carbon source determines that of the Bcy1 regulatory subunit; in addition, Bcy1 apparently determines the localization of the TPK subunit associated with it [81–83]. Nuclear accumulation of the regulatory Bcy1 subunit when glucose becomes available may favor the rapid recover of mitotic growth, whereas in cells growing on a non-fermentable carbon source or upon glucose exhaustion it may be advantageous to increase the cytoplasmic level of Bcy1 in order to downregulate the PKA signal, thus promoting the switch from a fermentative to a respiratory/gluconeogenic metabolism or the entry into quiescence [81]. Finally, several evidences [84] suggest that the TOR network controls the subcellular localization of both the *TPK1* catalytic subunit and the Yak1 kinase that affect Bcy1 localization through multiple phosphorylations of its N- terminus [82].

Activation of PKA elicits dramatic changes in the transcriptional program and in the activity of the biosynthetic machinery, which help the yeast cells to adapt to changes in the nutrient status. Several well known targets of PKA include glycolytic and gluconeogenetic enzymes, proteins involved in the metabolism of storage carbohydrates, transcription factors regulating stress response, ribosomal biogenesis, and carbohydrate metabolism [59,85]; see also Section 3.2.3.

#### 3.2.1. The GPCR System

The GPCR (G-protein coupled receptor) module composed by the Gpr1 receptor and its cognate G protein Gpa2 defines a glucose-sensing system that works in parallel with Ras to activate PKA (Figure 3) [59,60,62]. *GPR1* encodes a seven-transmembrane G protein–coupled receptor that physically interacts with Gpa2 [86,87], a small GTP-binding protein homologous to the mammalian Gα subunit of the heterotrimeric G proteins [88]. Binding of glucose to Gpr1 directs the formation of the GTP-bound, active form of Gpa2, which then stimulates adenylate cyclase to increase cAMP production [87].

The Gpr1-Gpa2 module is responsive to glucose and sucrose but not to structurally similar sugars such as fructose or to glucose analogues, while mannose acts as a potent antagonist of both sucrose and glucose induced cAMP signaling [89,90]. Although no direct binding of any sugar to Gpr1 has been reported so far, indirect mutational evidence supports the existence of a sucrose and glucose binding site(s) in Gpr1 [90], Figure 2.

Gpr1 senses sucrose and glucose with high (0.5 mM) and low (20 mM) affinity, respectively. Detection of low amounts of sucrose, a less-preferred sugar, may be important for the survival of yeast cells in the wild, where long periods of nutrient starvation alternate with intervals of nutrient abundance. On the other hand, the low affinity of the GPCR system for glucose may fit with the notion that its major function is confined to cAMP synthesis stimulation during the transition from respirative growth on a non-fermentable carbon source to fermentative growth on glucose [62,90,91]. The complete switch from a respirative/gluconeogenetic metabolism to fermentative growth in fact occurs at *ca.* 20 mM glucose [62,90,90], a concentration close to the apparent *Ka* estimated for glucose activation of cAMP synthesis.

*GPA2* deletion confers to some extent the typical phenotype associated with reduced PKA activity [87], *GPA2* or *GPR1* inactivation delaying several PKA-controlled processes (such as the mobilization of the reserve carbohydrates, loss of heat resistance, repression of STRE responsive genes, and induction of genes encoding ribosomal proteins) that occur during the transition to growth on glucose [71,87]. *GPA2* or *GPR1* deletion also affects cell size of glucose-grown (but not ethanol-grown) cells [93–95]; see Sections 7.3 and 8. The GPCR module is not required for intracellular acidification-induced PKA activation and does not play a major role in controlling the basal cAMP level [71,87].

Gpa2 is an atypical Gα protein for which no canonic Gβ and Gγ cognate subunits have been identified. Recent findings suggest that Asc1 (a protein with no homology to the classical Gβ subunits but which possesses their characteristic 7-WD domain structure) may act as a substitute Gβ subunit for Gpa2 to negatively regulate the glucose signalling [96]: in fact, Asc1 binds directly to the inactive GDP-bound form of Gpa2 and inhibits guanine nucleotide exchange activity on Gpa2. In addition, Asc1 interacts with the adenylyl cyclase enzyme (Cyr1) and diminishes the cAMP production following glucose stimulation [96]. No putative Gγ subunits that bind to Asc1 have been identified so far.

Gpa2 also interacts with Krh1 and Krh1, two kelch repeat proteins originally thought to mimic Gβ subunits [97,98] but whose function is apparently to downregulate the PKA pathway [99–102]: it has been proposed that the kelch protein may facilitate the association between the regulatory (Bcy1) and catalytic (Tpk1,2,3) subunits of PKA [101,102]; as an alternative, Krh1/2 may inhibit the Ras signaling by increasing the levels of Ira1,2 [100].

Based on its interaction with the Kelch protein Krh1 and Krh2, Gpg1 was initially considered as the potential Gγ subunit in the putative heterotrimeric G-protein comprising Gpa2 and Krh1/2 as α and β subunits, respectively [96]. Current experimental evidence suggests for Gpg1 a role as an activator of the PKA pathway [59,96]. In addition, Gpa2 also binds to Rgs2, a protein that may functions as negative regulator of the GPCR system by stimulating the intrinsic GTPase activity of Gpa2 [103].

#### 3.2.2. Interdependence between the GPCR System, Ras and Glucose Phosphorylation in Glucose-Induced cAMP Signaling

Glucose- (or sucrose) dependent activation of cAMP signaling through the GPCR system is strictly dependent on sugar uptake and phosphorylation [89,104]. Addition of sucrose to an invertase deficient strain activates cAMP synthesis only if a low level of glucose is added so that glucose phosphorylation can be sustained [89].

The glucose transporters have no regulatory function, being only required to maintain a critical level of intracellular glucose to sustain sugar phosphorylation. [89]. In addition, neither of the two glucose sensors Snf3 and Rgt2 has a direct role in the cAMP signaling [104].

The constitutively active *GPA2^V132^* protein can fully substitute for the requirement of high extracellular glucose in cAMP signaling, allowing ligands that are phosphorylated but not detected by the Gpr1-Gpa2 system (such as fructose and low glucose) to fully activate the cAMP circuit [62,89]. Intracellular acidification can bypass the requirement for glucose uptake, but not for sugar phosphorylation. Neither glucose-6-phosphate nor ATP seem to act as “metabolic messengers” to trigger the cAMP production in response to glucose, since there is no strict correlation between the increase of these metabolites after glucose addition and the amplitude of the cAMP signal. Thus, since no further glucose metabolism is needed beyond glucose phosphorylation to activate the cAMP synthesis, a regulatory role for the sugar kinases has been proposed [104].

Hence, glucose-induced cAMP signaling clearly involves two distinct processes: an extracellular glucose-sensing process that is dependent on Gpr1-Gpa2 system and an intracellular glucose-sensing process that is dependent on glucose phosphorylation [62,91]. It is unclear why glucose phosphorylation is required and how it is coupled to the control of cAMP synthesis [62,85]. Glucose phosphorylation seems to be required to make adenylate cyclase responsive to activation by the GPCR system [73,91].

Interestingly, glucose-induced Ras-GTP loading is also dependent on sugar uptake and phosphorylation, while it does not require the presence of a functional GPCR system [73]. The exact mechanisms by which glucose triggers Ras activity is still uncertain: no sugar-sensing system has yet been identified that could function as an upstream activator of Cdc25 to transmit the glucose signal to the Ras proteins [89]; indeed, several evidences suggest that Cdc25 may not itself be the signal receiver for glucose induced cAMP response [105]. The increase in Ras2-level in response to glucose may be mediated through inhibition of Ira proteins [73], confirming early reports which assigned to Ras a decisive role in the glucose-induced cAMP signaling [106–110].

#### 3.2.3. Downstream Elements of the cAMP/PKA Pathway

When glucose is available, activation of the cAMP/PKA pathway favors rapid growth and cell proliferation by stimulating the glycolytic flux and by repressing the stress response and the expression of genes required for respiratory metabolism [21,59,85,111,112]. Consistent with the key role of cAMP signaling in promoting fermentation, many of the identified targets of PKA are enzymes involved in carbon and energetic metabolism [59,85].

Glucose dependent activation of the PKA circuit promotes growth (mass accumulation) and a drastic increase in the cellular biosynthetic capacity by inducing the transcription of genes involved in ribosome biogenesis [113–116]. The PKA dependent activation domain of several genes encoding ribosomal proteins maps to Rap1 binding sites [114,115]. The subcellular localization of the zinc-finger transcription factor Sfp1, the master regulator of ribosome biogenesis (Ribi) and ribosomal protein (RP) genes, is regulated by both the cAMP/PKA and TOR network in response to nutritional and stress inputs [113,117,118]. Further details on PKA and TOR-dependent regulation of cell growth can be found in Section 7.

Nutrient availability, growth rate and stress response are intimately interconnected and the switch to fermentative metabolism coincides with downregulation of the stress response [59,61]. Two zinc-finger transcription factors, Msn2 and Msn4, mediate cAMP/PKA-dependent, glucose-triggered effects on repression of stress responsive genes [119–122]. Msn2 and Msn4 promote general stress-response by binding to stress responsive elements (STRE) in the promoter of their targets genes [119,123] in response to starvation for glucose (and other nutrients) and to a wide variety of stress conditions [119,122,124,125]. The Msn2/4 regulon includes genes encoding molecular chaperones, antioxidant proteins, enzymes involved in carbohydrate metabolism and proteolysis [122,124,125].

PKA apparently regulates processes such as growth, glycogen accumulation and stress response by suppressing Msn2/4-mediated gene expression [121]. Accumulation of Msn2/4 in the nucleus - a major step for regulation of Msn2/4 activity and subsequent activation of stress response - takes place at the level of subcellular localization controlled antagonistically by stress conditions and by several nutrient sensing networks (PKA, TOR; Snf1) [120,121,126–128]. Both transcription factors are predominantly cytoplasmic during exponential growth, whereas they rapidly concentrate in the nucleus in stressed cells or when nutrients such as glucose or nitrogen are depleted [120]. Msn2/4 activity can also be regulated at the level of DNA binding [129], transactivation [130], protein stability [131–134]. Full activation of Msn2/4 upon glucose starvation requires an additional mechanism - likely occurring after DNA binding -involving the Yak1 kinase [135], a negative growth regulator that antagonizes the cAMP/PKA pathway [136–139].

*YAK1* transcription is abolished in a *msn2 msn4* strain and correlates with cell cycle arrest [120,121,126–128,136]. The glucose-dependent regulation of Yak1 takes place at the level of subcellular localization [139]. In addition to the cAMP/PKA pathway, the TOR network also regulates intracellular distribution of Yak1 in response to nutritional and stress conditions [84,139,140]. Yak1 inhibits growth and stimulates the stress response, possibly by downregulating PKA activity: Bcy1, the regulatory subunit of yeast PKA, is phosphorylated and redistributes from nucleus to the cytoplasm in a Yak1-dependent manner upon glucose exhaustion. Intriguingly, PKA appears to control the localization of its own regulatory Bcy1 subunit via negative regulation of Msn2 and Msn4: low cAMP/PKA signalling activates the two transcription factors, leading to enhanced Yak1 transcription and increased cytoplasmic distribution of Bcy1 [82]. Yak1 has been found to stabilize or promote translation of mRNA encoding proteins involved in stress response, use of alternate carbon sources, growth inhibition and entry into stationary phase by direct phosphorylation of Pop2, a RNAse member of the Ccr4-Caf1-NOT deadenylation complex [139]. Crf1 (a co-repressor of transcription of genes encoding ribosomal proteins) is another target of Yak1 as well as of the TOR pathway (see 7.2).

In the absence of Msn2/4, PKA can still regulate several stress responsive genes such as *HSP12* and *HSP26* (encoding two small heat-shock proteins) by negatively modulating the activity of Hsf1 [141], a transcription factor controlling the expression of a large battery of genes involved in processes such as heat-stress response, protein folding and degradation, detoxification, energy generation, carbohydrate metabolism and cell wall organization [142–147]. The PKA-coordinated regulation of Msn2/4 and Hsf1 *via* Yak1 may be part of a mechanism to ensure proper balance between cell growth and stress adaptation in response to frequent changes in environmental conditions. Apparently, Yak1-dependent activation of Hsf1 and Msn2/4 is regulated by PKA but not by the TOR network [135].

Hsf1 may be regulated by stress-specific differential phosphorylation events, which affect its DNA binding activity [148]. PKA represses Hsf1 activity mostly through inhibition of Hsf1 phosphorylation by Yak1, which phosphorylates (and activates) Hsf1 when PKA activity decreases upon acute glucose starvation. Furthermore, although both Snf1 and Yak1 activate Hsf1 in response to glucose limitation, the two kinases are apparently involved in the adaptation to different physiological conditions: in particular, Yak1 (but not Snf1) seems to be primarily responsible for the activation of Hsf1 under acute glucose starvation. Snf1 and Yak1 phosphorylate different sites on Hsf1 that may result in transcription of distinct subset of genes depending on the sequence of HSE motifs [135].

In contrast to the role of PKA in the regulation of Msn2/4 dependent transcription, which affects all of STRE-containing genes, PKA activity influences only a subset of Hsf1 targets [141]. Despite a limited overlap between their target genes [124,125,149–151] Hsf1 and Msn2/4 may play distinct roles to ensure cell survival and growth recovery upon exposure to extreme temperatures [152].

PKA dependent phosphorylation negatively regulates the activity of Rim15, a critical kinase for entry into quiescence [153–155]. *rim15* null mutants fail to properly arrest in G0 upon nutrient exhaustion and exhibit decreased accumulation of storage carbohydrates, reduced expression of stress responsive genes and diminished thermotolerance. Rim15 likely inhibits expression of genes required for growth: consistently, inactivation of *RIM15* suppresses the lethality of *tpk1 tpk2 tpk3* triple null strain, whereas overexpression of Rim15 during exponential growth inappropriately elicits several stationary phase responses and causes a synthetic growth defect in mutants with reduced PKA activity [153–154]. Nuclear/cytoplasmic distribution of Rim15 is regulated by TOR (which responds to nitrogen source), Sch9, and by phosphate-responsive signaling complex Pho80/Pho85 [155–157]. Thus, at least three distinct nutrient-responsive pathways (cAMP/PKA (carbon source), TOR (nitrogen source) and Pho80/Pho85 (phosphate)) converge on Rim15. The effects of Rim15 on quiescence are due in part to the reconfiguration of the transcriptional profile, mediated through the stress response transcription factors Msn2/Msn4 and the related post diauxic shift transcription factor Gis1 [158,159]. Rim15 also binds to the Tps1 component of the trehalose synthase complex, suggesting that part of its role in quiescence involves direct regulation of key enzymatic activities [153,154].

A particularly relevant substrate of PKA is the transcriptional repressor Rgt1 [33], a key component of the Snf3/Rgt2 pathway which controls the expression of the major sugar transporters encoded by the *HXT* genes [7]; see 2.

## 4. Sensing Intracellular Glucose

Glucose represses the expression of a large number of genes, including those involved in the utilization of alternative carbon sources, gluconeogenesis and respiration through a process known as “glucose repression” [59,60,160–162]. This mechanism involves not only the repression of transcription when glucose is available, but also the release from the glucose-repressed state when the sugar becomes limiting. The signal for glucose repression requires glucose phosphorylation [59,60,162], consistently with the major –although enigmatic- role in glucose repression played by hexokinase 2 (Hxk2), the enzyme primarily responsible for catalyzing the first step of glycolysis when glucose is abundant [85,163–166]. No further sugar metabolism is required, since 2-deoxyglucose (a glucose analogue that can be phosphorylated but not further metabolized), is able to trigger repression [62].

The level of glucose repression correlates well with the sugar-transport capacity (the diverse hexose carriers do not have a specific regulatory role in the process [8,18,45,167,168] and the glycolytic flux rate. Even in high glucose media, glucose repression is fully operative only when cell posses sufficient glucose transport capacity to achieve a high glycolytic flux [168].

### 4.1. The Snf1 Signaling Transduction Pathway

#### 4.1.1. The Snf1 Protein Kinase Complex and Its Regulation

A central component in the signaling pathway for the glucose repression is the Snf1 kinase [162]. The Snf1 protein kinase complex [59,60], the yeast homologous of mammalian AMPK [169] is able to reprogram transcription of metabolic genes required for growth upon glucose exhaustion [162]. It also plays a role in chromatin remodeling and stress adaptation [162,170]. Snf1 complex is a heterotrimer and is composed of an α-subunit (Snf1) which contains a canonical kinase domain in its N-terminus and an auto-inhibitory domain in its C-terminus. The β-subunit (Sip1, Sip2, and Gal83, alternatively) regulates the subcellular localization [171] and the γ-subunit (Snf4) is required both for counteracting Snf1 auto-inhibition by the C-terminal regulatory domain and for glucose-regulated Snf1 phosphorylation on T210 [172]; see below.

Contrary to AMP kinase complex [59,60,162], Snf1 is not allosterically activated by AMP, although its activity correlates remarkably well with the AMP:ATP ratio, which rapidly increases upon glucose removal [62,173,174].

In accordance with its central role in adaptation to glucose depletion and utilization of alternative carbon sources, Snf1 is activated in response to glucose limitation. However, the actual signals triggering its activity have not yet been identified [162].

In presence of high glucose concentration, the regulatory domain of the *a*-subunit Snf1 binds to the catalytic domain, maintaining Snf1 in an auto-inhibited conformational state. When glucose is exhausted, Snf4 counteracts auto-inhibition of Snf1 by interacting with its regulatory domain and triggering a conformational change that results in Snf1 activation [162]. The key event in the entire process is the phosphorylation of a conserved threonine (T210) in the activation loop of Snf1 by one of three upstream kinases, encoded by *SAK1, ELM1*, and *TOS3* [175–177]. Snf1T210A mutation impairs Snf4 binding and prevents the activation of Snf1 [178,179]; in addition, the T210A substitution alters the subcellular localization of Snf1, blocking the nuclear accumulation of the kinase that occurs after a nutritional shift from high to low glucose [180]. The three upstream kinases are redundant in their Snf1-activating capacity, although Sak1 seems to play the prominent role in the regulatory process [175–177]. Tos3 involvement in Snf1 activation depends on carbon source availability since it plays a more active role during growth on non-fermentable carbon sources. Interestingly, Tos3 is a direct target of the activated Snf1 [181].

The type 1 protein phosphatase complex, comprising the Glc7 catalytic subunit and the Reg1 regulatory subunit, counteracts the activation of Snf1 mediated by the upstream kinases [179,182,183]. When a large supply of glucose becomes available, Reg1 interacts with the kinase domain of the active Snf1 complex and directs Glc7 to the activation loop of Snf1, resulting in T210 dephosphorylation and subsequent inactivation of Snf1 [184]. In *reg1* cells the Snf1 catalytic activity is constitutive and resistant to glucose inhibition [177].

Reg1 expression, localization, and interaction with Glc7 do not appear to be carbon source–modulated, but the activity of the Reg1/Glc7 complex may be regulated post-translationally: Reg1 is phosphorylated in a Snf1 dependent manner in response to glucose deprivation, whereas a rapid dephosphorylation of Reg1 occurs (likely mediated by Glc7) when sugar is added back to the growth medium [162,183]. Phosphorylation of Reg1 by Snf1 stimulates both the Glc7 mediated inactivation of Snf1 and the release of Reg1/Glc7 from its association with the Snf1 kinase complex. Interestingly, Reg1 phosphorylation appears to be regulated also by Hxk2, the major enzyme involved in the glucose repression pathway. Hxk2 may facilitate the inactivation of the Snf1 complex by the Glc7-Reg1 phosphatase, either by stimulating binding and/or phosphorylation of Reg1 or by inhibiting dephosphorylation of Reg1 by Glc7 [183].

Several lines of evidence suggest that the Snf1 upstream activating-kinases are not regulated by changes in the glucose levels [162,177]. In contrast, the dephosphorylation of the Snf1 activation loop is strongly stimulated in presence of high glucose level. However, the activity of the Glc7/Reg1 phosphatase does not appear to be directly influenced by glucose, since the Glc7/Reg1 enzyme seems to be equally active in both high and low glucose [185].

The activity of the Snf1 complexes is also regulated through a β-subunit–dependent subcellular localization [162]. During growth in glucose media, all the Snf1 complexes localize in the cytoplasm, regardless of the β-subunit [171]. When glucose becomes limiting, the β subunits (and their associated complexes) show unique subcellular localization patterns: Sip2-containing complexes are in the cytoplasm, while Sip1 and Gal83 complexes localize to the vacuolar membrane and to the nucleus, respectively. Gal83 contains a leucine-rich nuclear export signal (NES) in its N-terminus and its export depends on the Crm1 export receptor. Nuclear accumulation of Gal83-Snf1 requires also a functional kinase Sak1 [186]. The Snf4 subunit has both cytoplasmic and nuclear localization [171].

#### 4.1.2. Downstream Effectors of the Snf1 Protein Kinase Complex: Transcriptional Control in Response to Glucose Limitation

Snf1 regulates the expression of a large number of genes, including those involved in the metabolism of alternative carbon sources, gluconeogenesis, respiration, glucose transport, and meiosis. Early transcriptomic analysis showed that as many as 500 genes are modulated either directly or indirectly through a Snf1-dependent mechanism in response to glucose depletion [187–189]. A consistent fraction of Snf1 regulated genes is involved in transcription and signal transduction processes, reflecting the central regulatory role of this kinase [187]. However, only 10% of genes that show alteration of their expression profile in a *snf1* strain are direct targets of transcription factors regulated by Snf1 [187,188]. Although it has been recently shown that Snf1 complex mediates the glucose signal mainly in cooperation with the glucose-responsive cAMP/PKA pathway, it also specifically regulates a significant branch of the glucose repression mechanism not subject to PKA regulation [21].

Snf1 affects the transcription of genes required for metabolism of alternative carbon sources (such as sucrose, galactose, and maltose) mainly by modulating the activity of the Mig1 transcription repressor [190–193]. Mig1 is a Cys2-His2 zinc finger protein that during growth on glucose binds to a GC-rich consensus sequence of the promoter of its target genes and represses their transcription by recruiting the general co-repressors Ssn6 and Tup1 [190,194]. In the absence of glucose, Snf1 phosphorylates Mig1 [190] inhibiting the repressor activity (likely by altering the Mig1-Ssn6-Tup1 interaction) and promoting its nuclear export through the Msn5 importin [195,196]. When glucose becomes available, Mig1 is dephosphorylated and re-enters the nucleus where it can repress the transcription of its target genes. However, nuclear export does not seem to be strictly necessary to inactivate Mig1, since genes like *GAL1* are normally derepressed in a *msn5* null strain during growth on ethanol, despite the constitutive presence of Mig1 in the nucleus [60]. Several evidences suggest that Mig1 acts as a repressor in association with Hxk2, the major yeast hexokinase which is part of a repressor complex located on the *SUC2* promoter [164–166]. At high glucose concentrations, the Hxk2-Mig1 interaction on the promoter of target genes may prevent Snf1-dependent phosphorylation (and consequent inactivation) of Mig1 in order to maintain the transcriptional repressed status. The role of Hxk2 in glucose repression may be confined to a subset of glucose-repressible genes [41,85,197].

Two additional Snf1-regulated repressors are Nrg1 and Nrg2, both containing the Cys2-His2 zinc finger motif. Both Nrg1 and Nrg2 interact with Snf1, although they are not phosphorylated by the kinase. Rather, Snf1 appears to modulate Nrg2 level and is required for Nrg1 function (reviewed in [60]).

The Adr1 transcription factor (containing a Cys2-His2 DNA binding domain), which promotes expression of genes required for ethanol metabolism and β-oxidation of fatty acids [187,188], is activated in a Snf1-dependent manner upon glucose exhaustion [59]. Adr1 is also negatively regulated by PKA during growth on glucose and the mechanism of Adr1 inhibition by PKA or activation by Snf1 remains unclear. Apparently, Snf1 promotes Adr1 binding to chromatin but not transcriptional activation [198]. Adr1 is also regulated by Reg1, since *REG1* deletion increases Adr1 protein level and leads to induction of several Adr1-regulated genes, such as *ADH2* [199].

Cat8 and Sip4 activate the expression of genes required for gluconeogenesis during growth in the absence of glucose by binding carbon source response elements (CSRE) [200–203]. Both Cat8 and Sip4 are phosphorylated by the Snf1-Gal83 complex [201,204–206]. *CAT8* transcription is inhibited by Mig1 whereas *SIP4* expression is upregulated by Cat8 [59].

Together with Glc7, Snf1 participates in the regulation of the stress-response transcription factor Msn2 [207,208]. Glucose depletion results in rapid Msn2 dephosphorylation by Glc7 followed by Msn2 nuclear accumulation and activation of STRE-driven genes. Concurrently, Snf1 kinase is rapidly activated, leading to Mns2 nuclear exclusion and inactivation, so concurring to long-term adaptation to carbon stress [208].

Finally, glucose availability regulates via Snf1 the phosphorylation state and nuclear accumulation of Gln3, a GATA transcription factor involved in nitrogen starvation which is also a target of the TOR network [209].

#### 4.1.3. The Role of Snf1 Complex in Cell Cycle regulation

In mammalian and in *D. melanogaster* cells, activated AMPK inhibits the G1 to S transition either by promoting the synthesis of the CDK-inhibitor p21 in mammalian cells [210–212] or by down-regulation of cyclin E in *Drosophila melanogaster* [213,214]. Contrary to what observed in multicellular organisms, recent results from our laboratory newly indicate that Snf1 positively regulates yeast cell cycle progression by promoting expression of *CLB5* mRNA. In fact, in cells growing in 2% glucose synthetic media, *SNF1* deletion yields a severe slow growth phenotype, a specific delay in *CLB5* transcription and in the execution of the G1 to S transition. Both the slow growth phenotype and the delayed G1 to S transition can be fully rescued by expression of the phosphomimetic (Snf1^T210E^) forms of Snf1. Expression of the non-phosphorylable (Snf1^T210A^) form rescues both phenotypes only partially, suggesting that the α-catalytic subunit of Snf1 has a *CLB5* transcription promoting activity which is partially independent from its phosphorylation on T210. Such basal activity is in keeping with recent literature data showing that the non-phosphorylatable form of Snf1 (Snf1^T210A^) is able to control the high affinity potassium uptake system [215] and to regulate *HSP26* transcription [216]. Moreover, Snf1 regulates the levels of glucose-6P and trehalose when not phosphorylated in 5% glucose [217]. Instead, when Snf1-T210 phosphorylation is required, expression of Snf1^T210A^ is unable to complement the *SNF1* deletion as in the sucrose non-fermenting phenotype [215,218].

**Figure 4.**
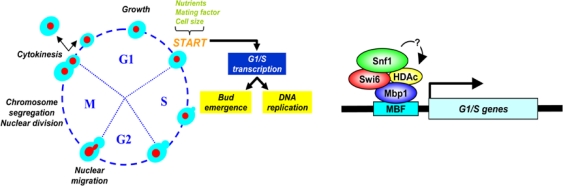
Snf1 is involved in control of MBF G1/S specific transcription factor. The left panel shows a simplified view of the yeast cell cycle showing some of its morphological and biochemical markers. The role of G1/S specific transcription is highlighted. The right panel shows known and putative interactions of Snf1 with proteins at MBF-dependent promoters. Not all proteins known to be bound at MBF-dependent promoters are shown. The Swi6 subunit of MBF transcription factor recruits the *a*-catalytic subunit of Snf1 to MBF-dependent promoter. Mbp1 and Swi6 form the MBF factor which binds the MBF-dependent promoters found upstream of genes involved in of the G1/S transition. A possible interaction of Snf1 with histone deacetylase (HDAC) at MBF-dependent promoters is shown, based on available evidence that mammalian AMPK and Snf1 can interact with histone acetylating/deacetylating enzymes (see text for details).

A specific interaction of Snf1 with Swi6, the transcription cofactor that forms complexes with DNA-binding proteins Swi4 and Mbp1 to regulate transcription at the G1/S transition, has been detected [Figure 4, 219]. Through its interaction with Swi6, Snf1 regulates expression of the *CLB5* mRNA, hence ensuring Clb5 protein accumulation and its activity on Sld2 phosphorylation [220], a necessary requirement for the onset of DNA replication and cell cycle [219,221]. Recently it was also shown that Snf1 directly controls Gcn5—a prototypic histone acetyltransferase, regulating transcription of various genes - most likely *via* direct interaction, since Snf1 overexpression suppresses phenotypes associated with expression of a phosphodeficient Gcn5 [222]. Thus our data fit well within an emergent view that links Snf1—acting as a transcriptional modulator-, chromatin remodelling complexes and G1/S-specific transcription that has been shown to undergo complex regulation through histone acetylation/deacetylation [223–225]. It is interesting to remember that activated mammalian AMPK has been shown to enhance SIRT1 deacetylase activity to promote transcriptional remodeling by sirtuins, explaining the convergent biological effects of AMPK on energy metabolism [226].

## 5. Interconnections among Glucose Sensing Mechanisms

The three glucose sensing pathways are intertwined in a complex regulatory network with multiple feedback and feedforward regulatory loops that serves to fine-tune the cellular response to glucose availability.

### 5.1. Integrated Regulation of HXT Expression

Multiple regulatory mechanisms ensure appropriate *HXT* gene expression. For example, the expression of *STD1*, one of the regulators of Rgt1 activity, is feedback regulated: glucose inhibits Std1 function by promoting its degradation by proteasome *via* the Rgt2/Snf3-Rgt1 signaling pathway [32] and concurrently induces *STD1* expression through the same pathway [34]. Thus, *STD1* expression is stimulated at the same time that Std1 protein levels are decreasing in response to glucose: this regulation might serve to dampen glucose induction of gene expression; moreover, it may also provide a mean for the rapid re-establishment of Rgt1-mediated repression upon glucose depletion [15,32]. Std1 may also play a role in the glucose repression pathway, since it is known to interact with and regulate Snf1 [38,52].

In contrast to *STD1*, its paralogue *MTH1*, which has an overlapping function, is feed-forward regulated: glucose reduces *MTH1* transcription *via* repression exerted by Mig1 and Mig2 while also stimulating the proteasome-mediated degradation of Mth1 [15,32,50,52,57]. Such a regulation reinforces the inhibitory effect of glucose on Mth1 function and ensures maximal glucose induction of Rgt1-repressed genes [34]. The different modulation of the two paralogs Mth1 and Std1 justifies their diverse role in assisting the Rgt1-mediated repression, with Mth1 being the primary regulator and Std1 serving to buffer the response to glucose [15,32,34,55].

The Snf3/Rgt2-Rgt1glucose induction pathway promotes expression of the Mig2 repressor [34], which cooperates with Mig1 (Snf1 pathway) in the glucose-induced repression of many genes [15,191–193]. *SNF3* transcription is repressed through Mig1 (Snf1 pathway) and Mig2 (Snf3/Rgt2 pathway) in presence of abundant glucose, probably reflecting the role of Snf3 as a sensor of low levels of sugar [7,13].

### 5.2. Snf1, Rgt2/Snf3 and Glucose Repression

Interestingly, two well-known players in the phenomenon of the glucose repression, Mig1 and Mig2, are differentially regulated, despite their largely overlapping functions: Mig1 (which has a prominent role in the repression process) is an effector of Snf1 which responds to intracellular signals generated by glucose metabolism and regulates the subcellular localization of Mig1 [60,85,166], whereas Mig2 (whose contribution to glucose repression is less relevant) is transcriptionally regulated by the Rgt2/Snf3-Rgt1 pathway [34].

Besides inhibiting the expression of several components of the Snf3/Rgt2-Rgt1 circuit (*i.e*., *MTH1* and *SNF3*), the Snf1-Mig1 pathway can also auto-regulate its own activity by repressing the transcription of *MIG1* through a mechanism involving Mig1 itself (in cooperation with Mig2) [34,191]: the effect of this auto-regulatory circuit is to mitigate the Mig1-mediated glucose repression, thus enabling a more rapid recovery from the repressed-state when the sugar is depleted.

Glucose dependent repression of several genes (*i.e.*, *SUC2*) is defective when the Mth1 co-repressor is lost [52] or cannot be degraded [25,32]. On the other hand, down-regulation of Snf1 activity in high glucose appears to be necessary for degradation of the Mth1 and Std1 co-repressors and the ensuing induction of the *HXT1* carrier [56]. Therefore, these observations imply a functional link between inactivation of Snf1 and degradation of Mth1 and Std1 [56,59]. Consistent with this proposal, glucose-induced degradation of Mth1/Std1 is prevented in strains where Snf1 is constitutively active (*i.e*., *reg1* and *hxk2* null strains) and in cells harboring a hyperactive *SNF1^G53R^* or overexpressing the *SAK1* kinase [13,56,227].

It is presently unknown how Snf1 inactivation in high glucose would promote degradation of Mth1 and Std1. A popular model for proteolytic removal of Mth1 and Std1 includes nuclear export of the corepressor, which must undergo phosphorylation by the membrane-tethered Yck1/2 prior to being ubiquitinated by SCF^Grr1^ [15,44,59,60,85]. Therefore, it has been proposed that Snf1 might regulate nuclear export of Mth1 and Std1: consistently, Mth1 and Std1 are nuclear in cells harboring active Snf1 [56]. However, a recent study has called into question the soundness of this view by demonstrating that Mth1 is apparently degraded inside the nucleus [58].

Recent evidences have shown that Yck1 and Yck2 casein kinase might respond to glucose signals from both the Rgt2/Snf3 circuit and the Glc7/Reg1 phosphatase complex (involved in the Snf1 pathway) to induce degradation of Mth1 and Std1 with the resultant expression of the hexose transporters and the hexokinase encoded by *HXK2*. Since both glucose transport and hexokinase participate in glucose metabolism necessary for activation of Glc7/Reg1, these observations highlight a new intriguing link between the Snf3/Rgt2 pathway and the Snf1 network [56,228].

### 5.3. Glucose Dependent Regulation of HXK2 Expression: Hints for a Possible Cross-Talk among the cAMP/PKA, Rgt2/Snf3 and Snf1 Pathways

As mentioned in Section 4.1.2., Hxk2 is a bifunctional protein: in the cytoplasm it works as a glycolytic enzyme, while in the nucleus it interacts with components repressing expression of several glucose-repressed genes [85,164–166].

Expression of *HXK2* is positively regulated by glucose availability, apparently through a complex crosstalk among the three major glucose sensing system: the Snf1 circuit, the cAMP/PKA network and the Snf2/Rgt2 pathway [80]. Transcriptional repression of *HXK2* in low glucose media involves Med8 and Rgt1. Med8—a subunit of the RNA polymerase II mediator complex which associates with core polymerase subunits to form the RNA polymerase II holoenzyme—binds constitutively to a DRS (downstream repressing sequence) found in the *HXK2* gene [80,229], while Rgt1 binds to its cognate element inside the *HXK2* promoter in a carbon source-dependent manner [80,230]. In low glucose, Snf1-dependent phosphorylation promotes Rgt1/Med8 interaction—a required event for repression of *HXK2* transcription—and DNA binding of Rgt1 to the *HXK2* promoter. In high glucose, Tpk3-dependent hyperphosphorylation of the repressor triggers its release from the *HXK2* promoter allowing Rgt1 interaction with nuclear Hxk2 [80]. See Section 4.1.2 for Hhk2/Mig1 interactions.

## 6. The Transcriptional Response to Glucose: Contributions of the Diverse Signaling Circuits

More than 40% of the genes in yeast genome change their expression levels by more than twofold within minutes following addition of glucose to yeast cells growing on a non-fermentable carbon source [21,111]: genes required for glycolysis, glucose uptake and ribosome biogenesis are up-regulated, whereas the transcription of genes involved in respiratory/gluconeogenetic metabolism, utilization of alternative carbon sources and stress response become repressed [21,111,112].

PKA, Snf1, Snf3/Rgt2-Rgt1 and heme-dependent transcriptional activators are responsible for the whole glucose-induced transcriptional response [21]. Transcriptomic analysis of mutants in the *RAS2* and *GPA2* genes indicate that the cAMP/PKA pathway—the Ras branch playing a more prominent role—is the main player in the transcriptional response to glucose [21,112]; other signaling pathways mediate a small fraction of the glucose signal, often in conjunction with PKA [21]. The Snf1 pathway mediates a significant portion of the glucose repression mechanism not subject to direct PKA control by regulating a small set of genes specialized in the metabolism of alternative carbon sources [21]; in addition, the Snf1 and the cAMP/PKA circuit cooperate in the regulation of several glucose-repressed genes [21,231]. Finally, in the presence of glucose the Snf3/Rgt2-Rgt1 pathway induces the expression of a small set genes required for sugar uptake, such as the *HXT* genes [21,34]. Several of the genes subject to regulation by the Snf3/Rtg2-Rgt1 circuit also respond to PKA activation and require PKA activity for full induction by glucose [7,21,33]. Despite the fact that both Sch9 (a member of the AGC family of kinases, that is the closest yeast homolog to the mammalian S6 kinase and prosurvival Akt/PKB) and PKA regulate a massive, nutrient-dependent reconfiguration of the transcriptional program to promote growth under favorable conditions, they are likely to do so in response to different nutritional cues [21,59,112].

About 25% of the glucose-repressed genes and 10% of the glucose-induced genes respond to sugar addition even in the absence of PKA activity, and this regulation depends on glucose import. Many of the glucose-responsive genes whose induction depend (at least partially) on sugar uptake are involved in cell cycle progression and contain MCB and SCB regulatory motifs (active at the G1/S transition) in their promoter. In contrast, genes which require glucose transport to be repressed are often involved in the oxidative metabolism (enzymes of the TCA cycle and electron transport chain) [112].

### 6.1. Nutrients, Transcriptional Profile and Growth Rate

Nutrient availability influences growth rate [232,233] and yeast cell adapt to nutrient availability by changing their transcriptional profile. Transcriptomic analysis of chemostat-grown yeast grown under six different nutritional limitations showed that expression of more than one quarter of all yeast genes is linearly correlated with growth rate, regardless of the limiting nutrient [234]: the expression level of some genes (such as ribosome biogenesis genes) is directly proportional to the growth rate, whereas that of others is inversely proportional. There is a considerable overlap between the transcriptional responses to growth limitation and a wide variety of environmental stresses: consistently, cells growing slowly are also cross protected against heat-shock [235].

Metabolite concentrations can regulate gene expression, which can in turn regulate metabolic activity. Recent analyses of the metabolomic and transcriptional responses of *S. cerevisiae* to carbon and nitrogen starvation indicate that transcripts and metabolites show coordinated response dynamics. Furthermore, metabolites and gene products whose concentration profiles are similar tend to participate in related biological processes [236]. Thus, the nutrient status appears to establish both the cellular growth rate and a corresponding highly distinctive transcriptional (and metabolic) profile [234–237].

## 7. Connecting Glucose Sensing and Availability to Cell Growth and Division

Apparently, a yeast cell adjusts its transcriptional program, metabolic machinery and growth rate solely on the basis of its perception of the nutrient status, not on the basis of metabolites actually produced from the available nutrients [21,112,238]. Under most conditions, this kind of regulation is quite efficient, since the nutrients which the cell recognizes as being present in its living environment are actually available. However, a mismatch between what cell perceives and the real nutrient status (as a result of drug treatment or genetic manipulation) can have dramatic consequences: consistent with this notion, strains with an hyper-active cAMP/PKA pathway cannot grow on non-fermentable carbon sources [61], since these cells perceive a rich nutritional environment that it does not exist [21].

While conventional growth models focus almost exclusively on glucose uptake and metabolism, more recent experiments clearly indicate that yeast growth rate is determined not only by glucose uptake, but by glucose sensing as well [239]. More specifically, growth rate is determined by the *interaction* between glucose sensing and import, not by their individual actions and can thus be configured as a system-level property [240]. Appropriately disrupting this interaction can significantly change the cell’s growth rate, even if glucose transport rate does not change.

### 7.1. PKA Signaling and Cell Growth

Glucose dependent activation of the PKA circuit promotes growth rate, mass accumulation and a substantial increase in the cellular biosynthetic capacity. Conversely, inactivation of the PKA signaling pathway causes first cycle arrest at START, a regulatory area in the G1 phase of the cell cycle, followed by entry into stationary phase (G0) [59,60,241]. Even when supplied with a rapidly fermentable carbon source, mutants with reduced PKA activity exhibit several characteristics typical of stationary phase cells, including enhanced stress resistance, high level of storage carbohydrates, impaired filamentous growth and enhanced sporulation efficiency. Conversely, mutants with upregulated PKA pathway grow poorly on non fermentable carbon source, are sensitive to various stress forms, do not arrest properly in stationary phase when deprived of nutrients, exhibit a vigorous filamentous growth, but fail to sporulate [59–62,91,241]. These phenotypes partially arise from the inability to activate a stress response, but also from the lack of stored nutrients (such as glycogen or trehalose) needed to complete a round of mitotic division cell cycle upon starvation [62,241,242]. Constitutive PKA activity would stimulate cells to use all of their resources for metabolic growth and the lack of nutritional reserves would make them vulnerable to sudden stressful conditions [242].

### 7.2. Connections with other Nutrient Sensing Pathways Pathway

#### 7.2.1. The TOR Pathway

In addition to the cAMP/PKA signaling cascade, the other major nutrient-responsive, growth-controlling pathway in yeast is the TOR network [243–246]. Tor (Target of rapamycin) serine/threonine kinases belong to the phosphatidylinositol-3 kinase (PI3K) family and exert their functions in two distinct multiproteic complexes [247,248]: TOR Complex 1 (TORC1), which control various aspects of yeast growth and cell proliferation, and TORC2, which regulates cell polarity and organization of the actin cytoskeleton (and will not be considered any further in this review). The two complexes are structurally and functionally conserved in all the eukaryotes [244].

TORC1 activity responds to the nutritional status, primarily the quality of the nitrogen source, and to a wide variety of stress conditions, apparently relaying amino acid concentrations, glucose, and perhaps other nutrient signals to the cellular machinery [244,246,249,250]. Its major function appears to be the regulation of translation capacity in response to environmental signals by promoting ribosome biogenesis, amino acid availability, and translation efficiency [59,243–245,251].

Inhibition of TORC1 by rapamycin (a macrolide drug that in complex with the prolyl-isomerase FKBP12 binds to TOR suppressing its interaction with target substrates) mimics nutrient starvation and causes G1 arrest, inhibition of protein synthesis, glycogen accumulation, induction of autophagy and entry into quiescence [244,252]. Rapamycin causes G1 arrest by a dual mechanism that comprises downregulation of the G1-cyclins Cln1-3 [252] and upregulation of the Cdk inhibitor protein Sic1 [253]. The increase of Sic1 level is mostly independent of the downregulation of the G1 cyclins, requires Sic1 phosphorylation of T173 and involves nuclear accumulation of a more stable, non-ubiquitinated protein. Either *SIC1* deletion or *CLN3* overexpression results in non-cell-cycle-specific arrest upon rapamycin treatment and makes cells sensitive to a sublethal dose of rapamycin and to nutrient starvation [253]. Under nutrient-rich conditions, TORC1 inhibits the activity of transcriptional factors involved in nitrogen catabolite-repression (Gat1, Gln3) [128,254], retrograde response (Rtg1, Rtg3; [255–257] and stress-response (Msn2, Msn4) [127], whereas it promotes the function of transcriptional regulators involved in ribosome biogenesis (Fhl1, Spf1) [113,117,140,243]. One common regulatory mechanism involves a TORC1-mediated change in the phosphorylation state of these transcription factors, which alters their subcellular localization: these phosphorylation/dephosphorylation events are often not performed directly by the TORC1 complex but instead are carried out by downstream effectors, such as the PP2A (protein phosphatase 2A) or PP2A-like phosphatase complexes or the kinase Yak1 [84,127,128,249,258,259]. The AGC kinase Sch9, the yeast equivalent of mammalian S6 kinase (S6K), directly mediates many of the TORC1-dependent effects on growth and mass accumulation [260].

Expression of the genes encoding the numerous constituents of ribosomes requires transcription by all three classes of nuclear RNA polymerase: TOR controls other aspects of ribosome biogenesis, such as the Pol I- and Pol III-dependent transcription of the rDNA and tRNA genes via phosphorylation of dedicated transcription factors [261]. Tor1 itself may activate rDNA transcription in rich nutrient conditions by entering the nucleus and binding directly to promoters [262]; however, in other studies, Tor1 has been localized to internal membrane structures but not the nucleus [248,263,264]. TORC1 is also intimately implicated in vesicular trafficking [250,265].

#### 7.2.2. TOR-PKA Connections

“Core growth related genes” (encoding ribosomal proteins and key metabolic enzymes) and stress related genes appear to be regulated independently by the two primary nutrients: carbon and nitrogen sources. Carbon source regulates the expression of these genes through the PKA pathway, whereas nitrogen source impinges on the expression of growth and stress related genes through the TORC1 pathway, which has been shown to directly regulate the activity of Sch9 [21,260]. Consistent with the proposal that the TOR and PKA signaling cascades independently coordinate the expression of genes required for growth and the stress response, the inhibition of TOR signaling by rapamycin results in repression of the RP genes and induction of the STRE genes, whereas mutations that hyperactivate the PKA circuit confer resistance to rapamycin and relieve the transcriptional repression of RP genes imposed by rapamycin [116]. By contrast, partial inactivation of the PKA signaling cascade enhances rapamycin sensitivity, but has only minor effects on RP gene expression. Complete loss of PKA function diminishes RP gene expression and concurrently up-regulates STRE gene expression; remarkably, this altered transcriptional profile is still sensitive to rapamycin and thus subject to TOR control [116].

However, the exact relationship between the TOR and PKA networks is still controversial. As an alternative model, it has been proposed that TOR may act upstream of Ras to regulate PKA activity [84,140]: according to Hall and co-workers, the Ras/PKA circuit would represent a distinct branch of the TOR network that would regulate gene transcription (in particular RP genes) independently from the Tap42/PP2A phosphatase and Sch9 route [84,140]. In support of this view, TOR appears to regulate the subcellular localization (and possibly the activity) of the catalytic Tpk1 subunit of PKA and of the Yak1 kinase [84]; see Section 3.2.3. Anyhow, many genes require both PKA and TOR for proper nutrient regulation and the concurrent inactivation of the PKA and TOR signaling (combined with loss of glucose transport activity) is sufficient to prevent virtually the entire transcriptional response to nutrients [112].

Notably, the subcellular localization of the zinc-finger transcription factor Sfp1, the master regulator of Ribi and RP genes, is regulated by both the cAMP/PKA and TOR network in response to nutritional and stress inputs. In actively growing cells, Sfp1 is found inside the nucleus, but it rapidly translocates into the cytoplasm in response to carbon and nitrogen starvation, oxidative stress, as well as inactivation of TOR signaling [113,117,118]. Recent evidences have demonstrated that Sfp1 is a direct substrate of the TORC1 complex, which regulates Sfp1 function via phosphorylation at multiple residues. Sfp1, in turn, negatively regulates TORC1 phosphorylation of Sch9, the other key target of TOR in the control of ribosome biogenesis, thus revealing a feedback mechanism that regulates RP and Ribi genes transcription [266].

An additional transcription factor that regulates RP and Ribi genes expression in response to TOR and PKA signaling is the forkhead-like protein Fhl1, together with its co-regulators Ifh1 and Crf1 [113,140,267–269]. Fhl1 has a dual role as an activator and a repressor in the transcription of ribosomal protein genes that is determined by its direct interactions with the coactivator Ifh1 and the corepressor Crf1. In growing cells, TOR maintains Crf1 inactive in the cytoplasm by repressing the Yak1 kinase, possibly via a PKA dependent route. When TOR is inactive, Yak1 directly phosphorylates Crf1, thus promoting the nuclear accumulation of the corepressor: once inside the nucleus, the phosphorylated Crf1 displaces Ifh1 from Fhl1 (which is constitutively bound to RP gene promoters), thereby inhibiting transcription of RP genes [140]. As an additional layer of regulation, the nuclear localization of both Fhl1 and Ifh1 is influenced by Sfp1 [113].

Many diverse environmental stresses [including heat shock, osmotic stress, oxidative stress and DNA damage) that activate Msn2/4 through decreased PKA or TOR signaling induce at least a transient arrest of cell cycle progression: Xbp1, a transcriptional repressor with homology to Swi4 and Mbp1, is induced by stress and glucose starvation and may contribute to repress the transcription of the G1 cyclins-encoding genes, thus causing a transient cell cycle delay under stress conditions [270–272]. Although the cAMP/PKA circuit affects ribosome biogenesis ([113] and previous sections), the impact of PKA inactivation on cell cycle progression is too rapid to be the simple result of diminished cellular biosynthetic capacity [59,273]. Transcriptomic analyses revealed several intriguing connections between the TOR/Sch9 network and the Gpr1/Gpa2 branch of the cAMP/PKA signaling cascade [21] that may play a decisive role in the developmental program that yeast cells adopt under nutrient shortage.

### 7.3. Nutritional and Genetic Modulation of Cell Size

For free living microorganism like the budding yeast *S. cerevisiae*, the capacity to regulate growth and cell cycle progression according to the nutrient availability is a major fitness factor: proliferation has to be rapid when large supplies of nutrients are available and has to stop when these becomes exhausted. For instance, it would, be deleterious for a yeast cell to engage in energetically expensive cellular processes or to attempt proliferation under unfavorable conditions. Nutrients like glucose must therefore generate signals that are somehow received and elaborated by the complex machinery governing growth and cell cycle progression.

In *S. cerevisiae* regulation of cell cycle progression is exerted predominantly during a narrow interval in the late G1 phase known as START [274]. At START a yeast cell integrates environmental and internal signals (such as nutrient availability, presence of pheromone, obtainment of a critical size, status of the metabolic machinery) and decides whether to enter a new cell cycle or to undertake an alternative developmental program (sporulation, pseudohyphal differentiation, entry into stationary phase). Execution of START irreversibly commits the cell to a new mitotic cycle and requires the activation of Cdk1, the cyclin-dependent kinase governing the major cell cycle transitions in budding yeast whose activity is regulated by its association with multiple regulatory subunits known as cyclins (see [275] for a recent review). Nutritional availability also modulates the degree of asymmetry of cell division: poor media usually yield large parent cells and very small daughters, whereas in rich media the asymmetry between parent and daughter cells is reduced (reviewed in [276]).

To maintain cell mass homeostasis, cell proliferation requires a precise coordination between growth and cell division [232,233,277–279]. It has been proposed that coordination of mass accumulation with cell cycle progression relies on a sizer mechanism, so that DNA replication and/or cell division start only when cells have reached a critical cell size that we refer to as Ps. In this way, tiny newborn cells will have to grow more than mother cells before being able to overcome the cell size checkpoint. Conversely, a larger cell will overcome the cell size checkpoint earlier than the “normal, average” cell. As a result, both small and large cells will stabilize cell size to the “normal, average” value. Ps—operationally defined as the protein content of cells at the onset of DNA replication [280]—is function of the growth rate, being constant at low and medium rates, while it increases and almost doubles at fast rates [93,281,282].

A molecular mechanism for the sizer mechanism has been proposed [93,283]. At the end of the previous cycle each newborn cell (parent or daughter) receives—with a given cell mass—a set amount of Far1, and Sic1, two cyclin-dependent kinase inhibitors, and of Whi5, an inhibitor of SBF and MBF transcription factors. At each growth rate, cells grow having a constant level of the cyclin Cln3 per unit mass during the G1 phase: therefore the amount of Cln3 is a measure of cell mass accumulation [284,285]. A first threshold, that is the actual cell sizer, detects the reaching of a given cell size, fairly similar at all growth rates, when the amount of Cln3—that parallels that of total protein—overcomes that of Far1. Another threshold—dependent on the first—is activated after a sizable period of time (in the order of 30–40 min). This time period is required to execute a sequence of biochemical activities that involve both Cln- and Clb-Cdk complexes leading to actual S phase onset. A very relevant step is that in which Cln3-Cdk1, released free after the overcoming of the first threshold, phosphorylates Whi5 so to reduce its binding to the transcription factors SBF/MBF that hence are able to activate S phase specific transcription. The timer length is fairly constant both at high and low growth rates and contributes to set the actual, measured P_S_, making its value larger at faster growth rates. The structure of the G_1_-to-S module is therefore that of a sizer (the Far1/Cln3 threshold) plus a timer (see [281] for further details). Other authors, using a completely different approach (single-cell imaging analysis of fluorescently labeled cells), reached the same conclusion on the structure of the yeast G1-to-S network [286].

A further insight derived from our model is that the setting of Ps is recognized as an emergent property of the entire G1 to S network [283]. In fact, both the growth rate and several players of the G1 to S network have been shown, by sensitivity analysis, to affect the setting of Ps, giving therefore also an explanation of the observed dependence of the value of Ps from the rate of growth [279,232].

The cAMP pathway is involved in the control of cell cycle progression [60,287–289] and nutritional modulation of the critical cell size required for entry into the S phase [95,273,290,291]. In unperturbed exponentially growing yeast populations addition of cAMP to the medium largely increases Ps. cAMP delays the G1-to-S transition in small cells, but it is ineffective on large parent cells [292]. The cAMP effect is largely due to repression of *CLN1* and *CLN2* transcription [293,294]. On the contrary, Cln3 is not inhibited by the cAMP signal and counteracts this inhibition of other Clns by mediating their growth-dependent expression [293].

Mutant cells with reduced cAMP signaling generally exhibit a consistent decrease in cell size. A *cdc25* temperature-sensitive mutant shows a smaller size than its isogenic wild type strain [290]. The carbon-source-dependent modulation of cell size is also lost in a strain expressing a truncated version of *CDC25* lacking the amino-terminal region or heterologous GEFs: these mutant exhibits nearly identical reduced size both in glucose and ethanol [295]. A *tpk1^w^* *tpk2 tpk3 bcy1* quadruple-null mutant, who possesses a weak constitutive PKA activity, also exhibits reduced cell volume [294,296]. In the presence of glucose, *gpr1* and *gpa2* single and double mutants strains display small size [93,94] and reduced protein synthesis rate [94], whereas no alteration is apparent during growth on ethanol [93,94]. The doubling time and the length of the budded phase in glucose are unaffected by inactivation of the GPCR system, consistent with the notion that signaling through this circuit specifically modulates the critical size required for budding and DNA replication. Gpr1 and Gpa2 are also required for the rapid adjustment of cell size in response to glucose [93]; see also 8.

In contrast, hyper-activation of the cAMP pathway results in dramatically large cells. *RAS2^V19^*, a constitutively activated allele of *RAS2*, increases cellular mass [293]. The deletion of both *PDE1* and *PDE2*, which encode 3′-5′-cyclic nucleotide phosphodiesterases increase the cellular cAMP content and cell volume [291]. Furthermore, these strains respond to exogenous cAMP by increasing their size in a dose-dependent fashion [29,294]. Inactivation of *BCY1* and *IRA2* also leads to increased cell size [291,297].

## 8. Conclusions

It is becoming increasingly clear that in yeast the signaling roles of glucose, and possibly of other nutrients as well, has equal dignity to its metabolic role, as indicated by the fact that 90% of glucose effects can be recapitulated by activation of the PKA pathway [21]. This major role of glucose sensing is apparent not only at the level of gene expression, but at the level of cell physiology as well. We could in fact show that partial inactivation of glucose sensing severely hampers nutritional modulation of cell growth and division in exponentially growing cultures [93]. Similarly, the early phases of an ethanol-glucose nutritional shift-up are altered in mutants with an altered Gpr1/Gpa2 pathway [93]. By imposing a glucose uptake rate independent of the sensed extracellular glucose level, Youk and van Oudenaarden [239] could similarly show that growth rate does not depend simply on glucose uptake, but rather on the *interaction* between glucose sensing and transport. Such a strong dependency on signaling makes yeast capable of applying a *feed-forward* strategy [298] to fit its growth rate to the environment, thus maximizing its flexibility in adapting to a changing environment.

As knowledge of the complete interactions between nutrient sensing, utilization and metabolism on one side and the molecular mechanism of cell growth and cell cycle increases, it will be possible to define a multi-level understanding of yeast growth and division. Through the emerging tools of modular systems biology [2–4,281] that make use of integrated, iterative cycles of post-genomic analyses, hypothesis-driven experiments, modeling and simulation, a quantitative understanding of the dynamic behaviour of a cell will be ultimately possible. Studies, tools and methodologies conducted in model organisms such as *S. cerevisiae* could then contribute to put stronger foundation on the systems biology approach to problems of applicative relevance including industrial and medical biotechnology.

## References

[1] Goffeau A., Barrell B.G., Bussey H., Davis R.W., Dujon B., Feldmann H., Galibert F, Hoheisel J.D., Jacq C., Johnston M., Louis E.J., Mewes H.W., Murakami Y., Philippsen P., Tettelin H., Oliver S.G. (1996). Life with 6000 genes. Science.

[2] Kitano H. (2002). Looking beyond the details: a rise in system-oriented approaches in genetics and molecular biology. Curr. Genet.

[3] Westerhoff H.V., Palsson B.O. (2004). The evolution of molecular biology into systems biology. Nat. Biotechno.

[4] Alberghina L., Westerhoff H.V. (2005). Systems Biology, Definitions and Perspectives. Topics in Current Genetics.

[5] Gray J.V., Petsko G.A., Johnston G.C., Ringe D., Singer R.A., Werner-Washburne M. (2004). Sleeping beauty: quiescence in Saccharomyces cerevisiae. Microbiol Mol. Biol. Rev.

[6] Boles E., Hollenberg C.P. (1997). The molecular genetics of hexose transport in yeasts. FEMS Microbiol. Rev.

[7] Ozcan S., Johnston M. (1999). Function and regulation of yeast hexose transporters. Microbiol Mol Biol Rev.

[8] Reifenberger E., Boles E., Ciriacy M. (1997). Kinetic characterization of individual hexose transporters of *Saccharomyces cerevisiae* and their relation to the triggering mechanisms of glucose repression. Eur J. Biochem.

[9] Reifenberger E., Freidel K., Ciriacy M. (1995). Identification of novel HXT genes in *Saccharomyces cerevisiae* reveals the impact of individual hexose transporters on glycolytic flux. Mol Microbiol.

[10] Wieczorke R., Krampe S., Weierstall T., Freidel K., Hollenberg C.P., Boles E. (1999). Concurrent knock-out of at least 20 transporter genes is required to block uptake of hexoses in Saccharomyces cerevisiae. FEBS Lett.

[11] Ozcan S., Dover J., Johnston M. (1998). Glucose sensing and signaling by two glucose receptors in the yeast *Saccharomyces cerevisiae*. EMBO J.

[12] Maier A., Völker B., Boles E., Fuhrmann GF. (2002). Characterisation of glucose transport in *Saccharomyces cerevisiae* with plasma membrane vesicles (countertransport) and intact cells (initial uptake) with single Hxt1, Hxt2, Hxt3, Hxt4, Hxt6, Hxt7 or Gal2 transporters. FEMS Yeast Res.

[13] Ozcan S., Johnston M. (1995). Three different regulatory mechanisms enable yeast hexose transporter (HXT) genes to be induced by different levels of glucose. Mol. Cell Biol.

[14] Ozcan S., Dover J., Rosenwald A.G., Wölfl S., Johnston M. (1996). Two glucose transporters in *Saccharomyces cerevisiae* are glucose sensors that generate a signal for induction of gene expression. Proc. Natl. Acad. Sci USA.

[15] Johnston M., Kim J.H. (2005). Glucose as a hormone: receptor-mediated glucose sensing in the yeast *Saccharomyces cerevisiae*. Biochem. Soc. Trans.

[16] Krampe S., Stamm O., Hollenberg C.P., Boles E. (1998). Catabolite inactivation of the high-affinity hexose transporters Hxt6 and Hxt7 of *Saccharomyces cerevisiae* occurs in the vacuole after internalization by endocytosis. FEBS Lett.

[17] Krampe S., Boles E. (2002). Starvation-induced degradation of yeast hexose transporter Hxt7p is dependent on endocytosis, autophagy and the terminal sequences of the permease. FEBS Lett.

[18] Ye L., Kruckeberg A.L., Berden J.A., van Dam K. (1999). Growth and glucose repression are controlled by glucose transport in *Saccharomyces cerevisiae* cells containing only one glucose transporter. J. Bacteriol.

[19] van Suylekom D., van Donselaar E., Blanchetot C., Do Ngoc L.N., Humbel B.M., Boonstra J. (2007). Degradation of the hexose transporter Hxt5p in *Saccharomyces cerevisiae*. Biol. Cell.

[20] Diderich J.A., Schepper M., van Hoek P., Luttik MA., van Dijken J.P., Pronk J.T., Klaassen P., Boelens H.F., de Mattos M.J., van Dam K., Kruckeberg A.L. (1999). Glucose uptake kinetics and transcription of HXT genes in chemostat cultures of *Saccharomyces cerevisiae*. J Biol. Chem.

[21] Zaman S., Lippman S.I., Schneper L., Slonim N., Broach J.R. (2009). Glucose regulates transcription in yeast through a network of signaling pathways. Mol. Syst. Biol.

[22] Ozcan S., Johnston M. (1996). Two different repressors collaborate to restrict expression of the yeast glucose transporter genes HXT2 and HXT4 to low levels of glucose. Mol. Cell Biol.

[23] Ye L., Berden J.A., van Dam K., Kruckeberg A.L. (2001). Expression and activity of the Hxt7 high-affinity hexose transporter of *Saccharomyces cerevisiae*. Yeast.

[24] Dlugai S., Hippler S., Wieczorke R., Boles E. (2001). Glucose-dependent and -independent signaling functions of the yeast glucose sensor Snf3. FEBS Lett.

[25] Schulte F., Wieczorke R., Hollenberg C.P., Boles E. (2000). The HTR1 gene is a dominant negative mutant allele of MTH1 and blocks Snf3- and Rgt2-dependent glucose signaling in yeast. J. Bacteriol.

[26] Liang H, Gaber RF. (1996). A novel signal transduction pathway in *Saccharomyces cerevisiae* defined by Snf3-regulated expression of HXT6. Mol. Biol. Cell.

[27] Diderich J.A., Schuurmans J.M., Van Gaalen M.C., Kruckeberg A.L., Van Dam K. (2001). Functional analysis of the hexose transporter homologue HXT5 in Saccharomyces cerevisiae. Yeast.

[28] Verwaal R., Paalman J.W., Hogenkamp A., Verkleij A.J., Verrips C.T., Boonstra J. (2002). HXT5 expression is determined by growth rates in *Saccharomyces cerevisiae*. Yeast.

[29] Verwaal R., Arako M., Kapur R., Verkleij A.J., Verrips C.T., Boonstra J. (2004). HXT5 expression is under control of STRE and HAP elements in the HXT5 promoter. Yeast.

[30] Greatrix B.W., van Vuuren H.J. (2006). Expression of the HXT13, HXT15 and HXT17 genes in *Saccharomyces cerevisiae* and stabilization of the HXT1 gene transcript by sugar-induced osmotic stress. Curr Genet.

[31] Ozcan S., Leong T., Johnston M. (1996). Rgt1p of *Saccharomyces cerevisiae*, a key regulator of glucose-induced genes, is both an activator and a repressor of transcription. Mol. Cell Biol.

[32] Kim J.H., Brachet V., Moriya H., Johnston M. (2006). Integration of transcriptional and posttranslational regulation in a glucose signal transduction pathway in *Saccharomyces cerevisiae*. Eukaryot Cell.

[33] Kim J.H., Johnston M. (2006). Two glucose-sensing pathways converge on Rgt1 to regulate expression of glucose transporter genes in *Saccharomyces cerevisiae*. J. Biol. Chem.

[34] Kaniak A., Xue Z., Macool D., Kim J.H., Johnston M. (2004). Regulatory network connecting two glucose signal transduction pathways in *Saccharomyces cerevisiae*. Eukaryot Cell.

[35] Hirayama T., Maeda T., Saito H., Shinozaki K. (1995). Cloning and characterization of seven cDNAs for hyperosmolarity-responsive (HOR) genes of *Saccharomyces cerevisiae*. Mol. Gen Genet.

[36] Tomás-Cobos L., Casadomé L., Mas G., Sanz P., Posas F. (2004). Expression of the HXT1 low affinity glucose transporter requires the coordinated activities of the HOG and glucose signalling pathways. J. Biol. Chem.

[37] Tomás-Cobos L., Viana R., Sanz P. (2005). TOR kinase pathway and 14-3-3 proteins regulate glucose-induced expression of HXT1, a yeast low-affinity glucose transporter. Yeast.

[38] Tomás-Cobos L., Sanz P. (2002). Active Snf1 protein kinase inhibits expression of the *Saccharomyces cerevisiae* HXT1 glucose transporter gene. Bio. Chem. J.

[39] Petit T., Diderich J.A., Kruckeberg A.L., Gancedo C., Van Dam K. (2000). Hexokinase regulates kinetics of glucose transport and expression of genes encoding hexose transporters in *Saccharomyces cerevisiae*. J. Bacteriol.

[40] Belinchón M.M., Gancedo J.M. (2007). Different signalling pathways mediate glucose induction of SUC2, HXT1 and pyruvate decarboxylase in yeast. FEMS Yeast Res.

[41] Belinchon M.M., Gancedo J.M. (2007). Glucose controls multiple processes in *Saccharomyces cerevisiae* through diverse combinations of signaling pathways. FEMS Yeast Res.

[42] Coons D.M., Vagnoli P., Bisson L.F. (1997). The C-terminal domain of Snf3p is sufficient to complement the growth defect of snf3 null mutations in *Saccharomyces cerevisiae*: SNF3 functions in glucose recognition. Yeast.

[43] Vagnoli P., Coons D.M., Bisson L.F. (1998). The C-terminal domain of Snf3p mediates glucose-responsive signal transduction in *Saccharomyces cerevisiae*. FEMS Microbiol Lett.

[44] Moriya H., Johnston M. (2004). Glucose sensing and signaling in *Saccharomyces cerevisiae* through the Rgt2 glucose sensor and casein kinase I. Proc. Natl. Acad. Sci. USA.

[45] Ozcan S. (2002). Two different signals regulate repression and induction of gene expression by glucose. J. Biol. Chem.

[46] Dietvorst J., Karhumaa K., Kielland-Brandt M.C., Brandt A. (2010). Amino acid residues involved in ligand preference of the Snf3 transporter-like sensor in *Saccharomyces cerevisiae*. Yeast.

[47] Kim J.H., Polish J., Johnston M. (2003). Specificity and regulation of DNA binding by the yeast glucose transporter gene repressor Rgt1. Mol. Cell Biol.

[48] Kim J.H. (2009). DNA-binding properties of the yeast Rgt1 repressor. Biochimie.

[49] Mosley A.L., Lakshmanan J., Aryal B.K., Ozcan S. (2003). Glucose-mediated phosphorylation converts the transcription factor Rgt1 from a repressor to an activator. J. Biol. Chem.

[50] Flick K.M., Spielewoy N., Kalashnikova T.I., Guaderrama M., Zhu Q., Chang H.C., Wittenberg C. (2003). Grr1-dependent inactivation of Mth1 mediates glucose-induced dissociation of Rgt1 from HXT gene promoters. Mol. Biol. Cell.

[51] Polish J.A., Kim J.H., Johnston M. (2005). How the Rgt1 transcription factor of *Saccharomyces cerevisiae* is regulated by glucose. Genetics.

[52] Schmidt M.C., McCartney R.R., Zhang X., Tillman T.S., Solimeo H., Wölfl S., Almonte C., Watkins S.C. (1999). Std1 and Mth1 proteins interact with the glucose sensors to control glucose-regulated gene expression in *Saccharomyces cerevisiae*. Mol. Cell Biol.

[53] Lafuente M.J., Gancedo C., Jauniaux J.C., Gancedo J.M. (2000). Mth1 receives the signal given by the glucose sensors Snf3 and Rgt2 in *Saccharomyces cerevisiae*. Mol. Microbiol.

[54] Lakshmanan J., Mosley A.L., Ozcan S. (2003). Repression of transcription by Rgt1 in the absence of glucose requires Std1 and Mth1. Curr. Genet.

[55] Sabina J., Johnston M. (2009). Asymmetric signal transduction through paralogs that comprise a genetic switch for sugar sensing in *S. cerevisiae*. J. Biol. Chem.

[56] Pasula S., Jouandot. D., Kim J.H. (2007). Biochemical evidence for glucose-independent induction of HXT expression in *Saccharomyces cerevisiae*. FEBS Lett.

[57] Spielewoy N., Flick K., Kalashnikova T.I., Walker J.R., Wittenberg C. (2004). Regulation and recognition of SCFGrr1 targets in the glucose and amino acid signaling pathways. Mol. Cell Biol.

[58] Pasula S., Chakraborty S., Choi J.H., Kim J.H. (2010). Role of casein kinase 1 in the glucose sensor-mediated signaling pathway in yeast. BMC Cell Biol.

[59] Zaman S., Lippman S.I., Zhao X., Broach J.R. (2008). How *Saccharomyces* responds to nutrients. Annu. Rev. Genet.

[60] Santangelo G.M. (2006). Glucose signaling in *Saccharomyces cerevisiae*. Microbiol Mol. Biol. Rev.

[61] Thevelein J.M., de Winde J.H. (1999). Novel sensing mechanisms and targets for the cAMP-protein kinase A pathway in the yeast *Saccharomyces cerevisiae*. Mol. Microbiol.

[62] Rolland F., Winderickx J., Thevelein J.M. (2002). Glucose-sensing and -signalling mechanisms in yeast. FEMS Yeast Res.

[63] Martegani E., Baroni M.D., Frascotti G., Alberghina L. (1986). Molecular cloning and transcriptional analysis of the start gene CDC25 of *Saccharomyces cerevisiae*. EMBO J.

[64] Damak F., Boy-Marcotte E., Le-Roscouet D., Guilbaud R., Jacquet M. (1991). SDC25, a CDC25-like gene which contains a RAS-activating domain and is a dispensable gene of *Saccharomyces cerevisiae*. Mol. Cell Biol.

[65] Tanaka K., Nakafuku M., Satoh T., Marshall M.S., Gibbs J.B., Matsumoto K., Kaziro Y., Toh-e A. (1990). S. cerevisiae genes *IRA1* and *IRA2* encode proteins that may be functionally equivalent to mammalian ras GTPase activating protein. Cell.

[66] Tanaka K., Nakafuku M., Tamanoi F., Kaziro Y., Matsumoto K., Toh-e A. (1990). IRA2, a second gene of *Saccharomyces cerevisiae* that encodes a protein with a domain homologous to mammalian ras GTPase-activating protein. Mol. Cell Biol.

[67] Toda T., Uno I., Ishikawa T., Powers S., Kataoka T., Broek D., Cameron S., Broach J., Matsumoto K., Wigler M. (1985). In yeast, RAS proteins are controlling elements of adenylate cyclase. Cell.

[68] Casperson G.F., Walker N., Bourne H.R. (1985). Isolation of the gene encoding adenylate cyclase in *Saccharomyces cerevisiae*. Proc. Natl. Acad. Sci. USA.

[69] Sass P., Field J., Nikawa J., Toda T., Wigler M. (1986). Cloning and characterization f the high-affinity cAMP phosphodiesterase of S. cerevisiae. Proc. Natl. Acad. Sci. USA.

[70] Nikawa J., Sass P., Wigler M. (1987). Cloning and characterization of the low-affinity cyclic AMP phosphodiesterase gene of *Saccharomyces cerevisiae*. Mol. Cell Biol.

[71] Colombo S., Ma P., Cauwenberg L., Winderickx J., Crauwels M., Teunissen A., Nauwelaers D., de Winde J.H., Gorwa M.F., Colavizza D., Thevelein J.M. (1998). Involvement of distinct G-proteins, Gpa2 and Ras, in glucose- and intracellular acidification-induced cAMP signalling in the yeast *Saccharomyces cerevisiae*. EMBO J.

[72] Rudoni S., Colombo S., Coccetti P., Martegani E. (2001). Role of guanine nucleotides in the regulation of the Ras/cAMP pathway in *Saccharomyces cerevisiae*. Biochim. Biophys. Acta.

[73] Colombo S., Ronchetti D., Thevelein J.M., Winderickx J., Martegani E. (2004). Activation state of the Ras2 protein and glucose-induced signaling in *Saccharomyces cerevisiae*. J. Biol. Chem.

[74] Toda T., Cameron S., Sass P., Zoller M., Wigler M. (1987). Three different genes in S. cerevisiae encode the catalytic subunits of the cAMP-dependent protein kinase. Cell.

[75] Toda T., Cameron S., Sass P., Zoller M., Scott J.D., McMullen B., Hurwitz M., Krebs E.G., Wigler M. (1987). Cloning and characterization of BCY1, a locus encoding a regulatory subunit of the cyclic AMP-dependent protein kinase in *Saccharomyces cerevisiae*. Mol. Cell Biol.

[76] Robertson L.S., Fink G.R. (1998). The three yeast A kinases have specific signaling functions in pseudohyphal growth. Proc. Natl Acad. Sci. USA.

[77] Robertson L.S., Causton H.C., Young R.A., Fink G.R. (2000). The yeast A kinases differentially regulate iron uptake and respiratory function. Proc. Natl Acad. Sci. USA.

[78] Pan X., Heitman J. (2002). Protein kinase A operates a molecular switch that governs yeast pseudohyphal differentiation. Mol. Cell. Biol.

[79] Chevtzoff C., Vallortigara J., Averet N., Rigoulet M., Devin A. (2005). The yeastcAMPprotein kinase Tpk3p is involved in the regulation of mitochondrial enzymatic content during growth. Biochim. Biophys. Acta.

[80] Palomino A., Herrero P., Moreno F. (2006). Tpk3 and Snf1 protein kinases regulate Rgt1 association with *Saccharomyces cerevisiae* HXK2 promoter. Nucleic Acids Res.

[81] Griffioen G., Anghileri P., Imre E., Baroni M.D., Ruis H. (2000). Nutritional control of nucleocytoplasmic localization of cAMP-dependent protein kinase catalytic and regulatory subunits in *Saccharomyces cerevisiae*. J. Biol. Chem.

[82] Griffioen G., Branduardi P., Ballarini A., Anghileri P., Norbeck J., Baroni M.D., Ruis H. (2001). Nucleocytoplasmic distribution of budding yeast protein kinase A regulatory subunit Bcy1 requires Zds1 and is regulated by Yak1-dependent phosphorylation of its targeting domain. Mol Cell Biol.

[83] Griffioen G., Thevelein J.M. (2002). Molecular mechanisms controlling the localisation of protein kinase A. Curr Genet.

[84] Schmelzle T., Beck T., Martin D.E., Hall M.N. (2004). Activation of the RAS/cyclic AMP pathway suppresses a TOR deficiency in yeast. Mol. Cell Biol.

[85] Gancedo J.M. (2008). The early steps of glucose signalling in yeast. FEMS Microbiol Rev.

[86] Xue Y., Batlle M., Hirsch J.P. (1998). GPR1 encodes a putative G protein-coupled receptor that associates with the Gpa2p Galpha subunit and functions in a Ras-independent pathway. EMBO J.

[87] Kraakman L., Lemaire K., Ma P., Teunissen A.W., Donaton M.C., Van Dijck P., Winderickx J., de Winde J.H., Thevelein J.M. (1999). A *Saccharomyces cerevisiae* G-protein coupled receptor, Gpr1, is specifically required for glucose activation of the cAMP pathway during the transition to growth on glucose. Mol. Microbiol.

[88] Nakafuku M., Obara T., Kaibuchi K., Miyajima I., Miyajima A., Itoh H., Nakamura S., Arai K., Matsumoto K., Kaziro Y. (1988). Isolation of a second yeast *Saccharomyces cerevisiae* gene (GPA2) coding for guanine nucleotide-binding regulatory protein: studies on its structure and possible functions. Proc. Natl. Acad. Sci. USA.

[89] Rolland F., De Winde J.H., Lemaire K., Boles E., Thevelein J.M., Winderickx J. (2000). Glucose-induced cAMP signalling in yeast requires both a G-protein coupled receptor system for extracellular glucose detection and a separable hexose kinase-dependent sensing process. Mol. Microbiol.

[90] Lemaire K., Van de Velde S., Van Dijck P., Thevelein J.M. (2004). Glucose and sucrose act as agonist and mannose as antagonist ligands of the G protein-coupled receptor Gpr1 in the yeast Saccharomyces cerevisiae. Mol. Cell.

[91] Rubio-Texeira M., Van Zeebroeck G., Voordeckers K., Thevelein J.M. (2010). *Saccharomyces cerevisiae* plasma membrane nutrient sensors and their role in PKA signalling. FEMS Yeast Res.

[92] Johnston M., Carlson M., Gene Expression Jones P.E., Broach J.R. (1993). The Molecular and Cellular Biology of the Yeast Saccharomyces.

[93] Alberghina L., Rossi R.L., Querin L., Wanke V., Vanoni M. (2004). A cell sizer network involving Cln3 and Far1 controls entrance into S phase in the mitotic cycle of budding yeast. J. Cell Biol.

[94] Tamaki H., Yun C.W., Mizutani T., Tsuzuki T., Takagi Y., Shinozaki M., Kodama Y., Shirahige K., Kumagai H. (2005). Glucose-dependent cell size is regulated by a G protein-coupled receptor system in yeast *Saccharomyces cerevisiae*. Genes Cell.

[95] Tamaki H. (2007). Glucose-stimulated cAMP-protein kinase A pathway in yeast *Saccharomyces cerevisiae*. J. Biosci. Bioeng.

[96] Zeller C.E., Parnell S.C., Dohlman H.G. (2007). The RACK1 ortholog Asc1 functions as a G-protein beta subunit coupled to glucose responsiveness in yeast. J. Biol. Chem.

[97] Harashima T., Heitman J. (2002). The Galpha protein Gpa2 controls yeast differentiation by interacting with kelch repeat proteins that mimic Gbeta subunits. Mol. Cell.

[98] Harashima T., Heitman J. (2005). Galpha subunit Gpa2 recruits kelch repeat subunits that inhibit receptor-G protein coupling during cAMP-induced dimorphic transitions in *Saccharomyces cerevisiae*. Mol. Biol. Cell.

[99] Lu A., Hirsch J.P. (2005). Cyclic AMP-independent regulation of protein kinase A substrate phosphorylation by Kelch repeat proteins. Eukaryot Cell.

[100] Harashima T., Anderson S., Yates J.R., Heitman J. (2006). The kelch proteins Gpb1 and Gpb2 inhibit Ras activity via association with the yeast RasGAP neurofibromin homologs Ira1 and Ira2. Mol. Cell.

[101] Peeters T., Louwet W., Geladé R., Nauwelaers D., Thevelein J.M., Versele M. (2006). Kelch-repeat proteins interacting with the Galpha protein Gpa2 bypass adenylate cyclase for direct regulation of protein kinase A in yeast. Proc. Natl. Acad. Sci. USA.

[102] Peeters T, Versele M, Thevelein JM. (2007). Directly from Galpha to protein kinase A: the kelch repeat protein bypass of adenylate cyclase. Trends Biochem. Sci.

[103] Versele M., de Winde J.H., Thevelein J.M. (1999). A novel regulator of G protein signalling in yeast, Rgs2, downregulates glucose-activation of the cAMP pathway through direct inhibition of Gpa2. EMBO J.

[104] Rolland F., Wanke V., Cauwenberg L., Ma P., Boles E., Vanoni M., de Winde J.H., Thevelein J.M., Winderickx J. (2001). The role of hexose transport and phosphorylation in cAMP signalling in the yeast *Saccharomyces cerevisiae*. FEMS Yeast Res.

[105] Goldberg D., Segal M., Levitzki A. (1994). Cdc25 is not the signal receiver for glucose induced cAMP response in S. cerevisiae. FEBS Lett.

[106] Mbonyi K., Beullens M., Detremerie K., Geerts L., Thevelein J.M. (1988). Requirement of one functional RAS gene and inability of an oncogenic ras-variant to mediate the glucose-induced cAMP signal in the yeast *Saccharomyces cerevisiae*. Mol. Cell Biol.

[107] Munder T., Kuntzel H. (1989). Glucose-induced cAMP signaling in Saccharomyces cerevisiae is mediated by the CDC25 protein. FEBS Lett.

[108] Van Aelst L., Boy-Marcotte E., Camonis J.H., Thevelein J.M., Jacquet M. (1990). The C-terminal part of the *CDC25* gene product plays a key role in signal transduction in the glucose-induced modulation of cAMP level in *Saccharomyces cerevisiae*. Eur. J. Biochem.

[109] Van Aelst L., Jans A.W.H., Thevelein J.M. (1991). Involvement of the *CDC25* gene product in the signal transmission pathway of the glucose-induced RAS-mediated cAMP signal in the yeast *Saccharomyces cerevisiae*. J. Gen Microbiol.

[110] Bhattacharya S., Chen L., Broach J.R., Powers S. (1995). Ras membrane targeting is essential for glucose signaling but not for viability in yeast. Proc. Natl. Acad. Sci. USA.

[111] Wang Y., Pierce M., Schneper L., Güldal C.G., Zhang X., Tavazoie S., Broach J.R. (2004). Ras and Gpa2 mediate one branch of a redundant glucose signaling pathway in yeast. PLoS Biol.

[112] Slattery M.G., Liko D., Heideman W. (2008). Protein kinase A, TOR, and glucose transport control the response to nutrient repletion in *Saccharomyces cerevisiae*. Eukaryot Cell.

[113] Jorgensen P., Rupes I., Sharom J.R., Schneper L., Broach J.R., Tyers M. (2004). A dynamic transcriptional network communicates growth potential to ribosome synthesis and critical cell size. Genes Dev.

[114] Klein C., Struhl K. (1994). Protein kinase A mediates growth-regulated expression of yeast ribosomal protein genes by modulating RAP1 transcriptional activity. Mol. Cell Biol.

[115] Neuman-Silberberg F.S., Bhattacharya S., Broach J.R. (1995). Nutrient availability and the RAS/cyclic AMP pathway both induce expression of ribosomal protein genes in *Saccharomyces cerevisiae* but by different mechanisms. Mol. Cell Biol.

[116] Zurita-Martinez S.A., Cardenas M.E. (2005). Tor and cyclic AMP-protein kinase A: two parallel pathways regulating expression of genes required for cell growth. Eukaryot Cell.

[117] Marion R.M., Regev. A., Segal E., Barash Y., Koller D., Friedman N., O’Shea E.K. (2004). Sfp1 is a stress- and nutrient-sensitive regulator of ribosomal protein gene expression. Proc Natl Acad Sci USA.

[118] Fingerman I., Nagaraj V., Norris D., Vershon A.K. (2003). Sfp1 plays a key role in yeast ribosome biogenesis. Eukaryot Cell.

[119] Martínez-Pastor M.T., Marchler G., Schüller C., Marchler-Bauer A., Ruis H., Estruch F. (1996). The *Saccharomyces cerevisiae* zinc finger proteins Msn2p and Msn4p are required for transcriptional induction through the stress response element (STRE). EMBO J.

[120] Görner W., Durchschlag E., Martinez-Pastor M.T., Estruch F., Ammerer G., Hamilton B., Ruis H., Schüller C. (1998). Nuclear localization of the C2H2 zinc finger protein Msn2p is regulated by stress and protein kinase A activity. Genes Dev.

[121] Smith A., Ward M.P., Garrett S. (1998). Yeast PKA represses Msn2p/Msn4p-dependent gene expression to regulate growth, stress response and glycogen accumulation. EMBO J.

[122] Boy-Marcotte E., Perrot M., Bussereau F., Boucherie H., Jacquet M. (1998). Msn2p and Msn4p control a large number of genes induced at the diauxic transition which are repressed by cyclic AMP in *Saccharomyces cerevisiae*. J. Bacteriol.

[123] Moskvina E., Schuller C.T., Maurer C., Mager W.H., Ruis H. (1998). A search in the genome of *Saccharomyces cerevisiae* for genes regulated via stress response elements. Yeast.

[124] Gasch A.P., Spellman P.T., Kao C.M., Carmel-Harel O., Eisen M.B., Storz G., Botstein D., Brown P.O. (2000). Genomic expression programs in the response of yeast cells to environmental changes. Mol. Biol. Cell.

[125] Causton H.C., Ren B., Koh S.S., Harbison C.T., Kanin E., Jennings E.G., Lee T.I., True H.L., Lander E.S., Young R.A. (2001). Remodeling of yeast genome expression in response to environmental changes. Mol Biol Cell..

[126] Görner W., Durchschlag E., Wolf J., Brown E.L., Ammerer G., Ruis H., Schüller C. (2002). Acute glucose starvation activates the nuclear localization signal of a stress-specific yeast transcription factor. EMBO J.

[127] Santhanam A., Hartley A., Duvel K., Broach J.R., Garrett S. (2004). PP2A phosphatase activity is required for stress and Tor kinase regulation of yeast stress response factor Msn2p. Eukaryot. Cell.

[128] Beck T., Hall M.N. (1999). The TOR signaling pathway controls nuclear localization of nutrient-regulated transcription factors. Nature.

[129] Hirata Y., Andoh T., Asahara T., Kikuchi A. (2003). Yeast glycogen synthase kinase-3 activates Msn2p-dependent transcription of stress responsive genes. Mol. Biol. Cell.

[130] Boy-Marcotte E., Garmendia C., Garreau H., Lallet S., Mallet L., Jacquet M. (2006). The transcriptional activation region of Msn2p, in Saccharomyces cerevisiae, is regulated by stress but is insensitive to the cAMP signalling pathway. Mol. Genet. Genomics.

[131] Durchschlag E., Reiter W., Ammerer G., Schuller C. (2004). Nuclear localization destabilizes the stressregulated transcription factor Msn2. J. Biol. Chem.

[132] Lallet S., Garreau H., Poisier C., Boy-Marcotte E., Jacquet M. (2004). Heat shock-induced degradation of Msn2p, a *Saccharomyces cerevisiae* transcription factor, occurs in the nucleus. Mol. Genet. Genomics.

[133] Lallet. S, Garreau H., Garmendia-Torres C., Szestakowska D., Boy-Marcotte E., Quevillon-Chéruel S., Jacquet M. (2006). Role of Gal11, a component of the RNA polymerase II mediator in stress-induced hyperphosphorylation of Msn2 in *Saccharomyces cerevisiae*. Mol. Microbiol.

[134] Bose S., Dutko J.A., Zitomer R.S. (2005). Genetic factors that regulate the attenuation of the general stress response of yeast. Genetics.

[135] Lee P., Cho B.R., Joo H.S., Hahn J.S. (2008). Yeast Yak1 kinase, a bridge between PKA and stress-responsive transcription factors, Hsf1 and Msn2/Msn4. Mol. Microbiol.

[136] Garrett S., Menold M.M., Broach J.R. (1991). The *Saccharomyces cerevisiae* YAK1 gene encodes a protein kinase that is induced by arrest early in the cell cycle. Mol. Cell Biol.

[137] Garrett S., Broach J. (1989). Loss of Ras activity in *Saccharomyces cerevisiae* is suppressed by disruptions of a new kinase gene, YAKI, whose product may act downstream of the cAMP dependent protein kinase. Genes Dev.

[138] Ward M.P., Garrett S. (1994). Suppression of a yeast cyclic AMP-dependent protein kinase defect by overexpression of SOK1, a yeast gene exhibiting sequence similarity to a developmentally regulated mouse gene. Mol. Cell Biol.

[139] Moriya H., Shimizu-Yoshida Y., Omori A., Iwashita S., Katoh M., Sakai A. (2001). Yak1p, a DYRK family kinase, translocates to the nucleus and phosphorylates yeast Pop2p in response to a glucose signal. Genes Dev.

[140] Martin D.E., Soulard A., Hall M.N. (2004). TOR regulates ribosomal protein gene expression via PKA and the Forkhead transcription factor FHL1. Cell.

[141] Ferguson S.B., Anderson E.S., Harshaw R.B., Thate T., Craig N.L., Nelson H.C. (2005). Protein kinase A regulates constitutive expression of small heat-shock genes in an Msn2/4p-independent and Hsf1p-dependent manner in *Saccharomyces cerevisiae*. Genetics.

[142] Hahn J.S., Hu Z., Thiele D.J., Iyer V.R. (2004). Genome-wide analysis of the biology of stress responses through heat shock transcription factor. Mol. Cell Biol.

[143] Eastmond D.L., Nelson H.C. (2006). Genome-wide analysis reveals new roles for the activation domains of the *Saccharomyces cerevisiae* heat shock transcription factor (Hsf1) during the transient heat shock response. J Biol Chem.

[144] Wiederrecht G., Seto D., Parker C.S. (1988). Isolation of the gene encoding the S. cerevisiae heat shock transcription factor. Cell.

[145] Smith B.J., Yaffe M.P. (1991). A mutation in the yeast heat-shock factor gene causes temperature-sensitive defects in both mitochondrial protein import and the cell cycle. Mol. Cell Biol.

[146] Zarzov P., Boucherie H., Mann C. (1997). A yeast heat shock transcription factor (Hsf1) mutant is defective in both Hsc82/Hsp82 synthesis and spindle pole body duplication. J. Cell Sci.

[147] Imazu H., Sakurai H. (2005). *Saccharomyces cerevisiae* heat shock transcription factor regulates cell wall remodeling in response to heat shock. Eukaryot Cell.

[148] Hahn J.S., Thiele D.J. (2004). Activation of the *Saccharomyces cerevisiae* heat shock transcription factor under glucose starvation conditions by Snf1 protein kinase. J. Biol. Chem.

[149] Boy-Marcotte E., Lagniel G., Perrot M., Bussereau F., Boudsocq A., Jacquet M., Labarre J. (1999). The heat shock response in yeast: differential regulations and contributions of the Msn2p/Msn4p and Hsf1p regulons. Mol. Microbiol.

[150] Amorós M., Estruch F. (2001). Hsf1p and Msn2/4p cooperate in the expression of *Saccharomyces cerevisiae* genes HSP26 and HSP104 in a gene- and stress type-dependent manner. Mol. Microbiol.

[151] Treger J.M., Schmitt A.P., Simon J.R., McEntee K. (1998). Transcriptional factor mutations reveal regulatory complexities of heat shock and newly identified stress genes in *Saccharomyces cerevisiae*. J. Biol. Chem.

[152] Yamamoto N., Maeda Y., Ikeda A., Sakurai H. (2008). Regulation of thermotolerance by stress-induced transcription factors in *Saccharomyces cerevisiae*. Eukaryot Cell.

[153] Reinders A., Burckert N., Boller T., Wiemken A., De Virgilio C. (1998). *Saccharomyces cerevisiae* cAMPdependent protein kinase controls entry into stationary phase through the Rim15p protein kinase. Genes Dev.

[154] Swinnen E., Wanke V., Roosen J., Smets B., Dubouloz F., Pedruzzi I., Cameroni E., De Virgilio C., Winderickx J. (2006). Rim15 and the crossroads of nutrient signalling pathways in *Saccharomyces cerevisiae*. Cell Div.

[155] Pedruzzi I., Dubouloz F., Cameroni E., Wanke V., Roosen J., Winderickx J., De Virgilio C. (2003). TOR and PKA signaling pathways converge on the protein kinase Rim15 to control entry into G0. Mol. Cell.

[156] Wanke V., Pedruzzi I., Cameroni E., Dubouloz F., De Virgilio C. (2005). Regulation of G0 entry by the Pho80-Pho85 cyclin-CDK complex. EMBO J.

[157] Roosen J., Engelen K., Marchal K., Mathys J., Griffioen G., Cameroni E., Thevelein J.M., De Virgilio C., De Moor B., Winderickx J. (2005). PKA and Sch9 control a molecular switch important for the proper adaptation to nutrient availability. Mol. Microbiol.

[158] Pedruzzi. I., Bürckert N., Egger P., De Virgilio C. (2000). *Saccharomyces cerevisiae* Ras/cAMP pathway controls post-diauxic shift element-dependent transcription through the zinc finger protein Gis1. EMBO J.

[159] Cameroni E., Hulo N., Roosen J., Winderickx J., De Virgilio C. (2004). The novel yeast PAS kinase Rim 15 orchestrates G0-associated antioxidant defense mechanisms. Cell Cycle.

[160] Vanoni M., Sollitti P., Goldenthal M., Marmur J. (1989). Structure and regulation of the multigene family controlling maltose fermentation in budding yeast. Prog. Nucleic Acid Res. Mol. Biol.

[161] Carlson M. (1999). Glucose repression in yeast. Curr. Opin. Microbiol.

[162] Hedbacker K., Carlson M. (2008). SNF1/AMPK pathways in yeast. Front Biosci.

[163] De Winde J.H., Crauwels M., Hohmann S., Thevelein J.M., Winderickx J. (1996). Differential requirement of the yeast sugar kinases for sugar sensing in establishing the catabolite-repressed state. Eur. J. Biochem.

[164] Ahuatzi D., Herrero P., de la Cera T., Moreno F. (2004). The glucose-regulated nuclear localization of hexokinase 2 in *Saccharomyces cerevisiae* is Mig1-dependent. J. Biol. Chem.

[165] Ahuatzi D., Riera A., Peláez R., Herrero P., Moreno F. (2007). Hxk2 regulates the phosphorylation state of Mig1 and therefore its nucleocytoplasmic distribution. J. Biol. Chem.

[166] Moreno F., Ahuatzi D., Riera A., Palomino C.A., Herrero P. (2005). Glucose sensing through the Hxk2-dependent signalling pathway. Biochem. Soc. Trans.

[167] Elbing K., Ståhlberg A., Hohmann S., Gustafsson L. (2004). Transcriptional responses to glucose at different glycolytic rates in Saccharomyces cerevisiae. Eur. J. Biochem.

[168] Otterstedt K., Larsson C., Bill R.M., Ståhlberg A., Boles E., Hohmann S., Gustafsson L. (2004). Switching the mode of metabolism in the yeast *Saccharomyces cerevisiae*. EMBO Rep.

[169] Mitchelhill K.I., Stapleton D., Gao G., House C., Michell B., Katsis F., Witters L.A., Kemp B.E. (1994). MammalianAMP-activated protein kinase shares structural and functional homology with the catalytic domain of yeast Snf1 protein kinase. J. Biol. Chem.

[170] Lo W., Duggan S.L., Emre N.C., Belotserkovskya R., Lane W.S. (2001). Shiekhattar R, Berger SL. Snf1-a histone kinase that works in concert with the histone acetyltransferase Gcn5 to regulate transcription. Science.

[171] Vincent O., Townley R., Kuchin S., Carlson M. (2001). Subcellular localization of the Snf1 kinase is regulated by specific beta subunits and a novel glucose signaling mechanism. Genes Dev.

[172] Momcilovic M., Iram S.H., Liu Y., Carlson M. (2008). Roles of the glycogen-binding domain and Snf4 in glucose inhibition of SNF1 protein kinase. J. Biol. Chem.

[173] Wilson W.A., Hawley S.A., Hardie D.G. (1996). Glucose repression/derepression in budding yeast: SNF1 protein kinase is activated by phosphorylation under derepressing conditions, and this correlates with a high AMP: ATP ratio. Curr. Biol.

[174] Johnston M. (1999). Feasting, fasting and fermenting. Glucose sensing in yeast and other cells. Trends Genet.

[175] Nath N., McCartney R.R., Schmidt M.C. (2003). Yeast Pak1 kinase associates with and activates Snf1. Mol. Cell. Biol.

[176] Sutherland C.M., Hawley S.A., McCartney R.R., Leech A., Stark M.J., Schmidt M.C., Hardie D.G. (2003). Elm1p is one of three upstream kinases for the *Saccharomyces cerevisiae* SNF1 complex. Curr. Biol.

[177] Hong S.P, Leiper F.C., Woods A., Carling D., Carlson M. (2003). Activation of yeast Snf1 and mammalian AMP-activated protein kinase by upstream kinases. Proc. Natl. Acad. Sci. USA.

[178] Jiang R., Carlson M. (1996). Glucose regulates protein interactions within the yeast SNF1 protein kinase complex. Genes Dev.

[179] Ludin K., Jiang R., Carlson M. (1998). Glucose-regulated interaction of a regulatory subunit of protein phosphatase 1 with the Snf1 protein kinase in *Saccharomyces cerevisiae*. Proc. Natl. Acad. Sci. USA.

[180] Hedbacker K., Hong S.P., Carlson M. (2004). Pak1 protein kinase regulates activation and nuclear localization of Snf1-Gal83 protein kinase. Mol. Cell Biol.

[181] Kim M.D., Hong S.P., Carlson M. (2005). Role of Tos3, a Snf1 protein kinase kinase, during growth of *Saccharomyces cerevisiae* on nonfermentable carbon sources. Eukaryot Cell.

[182] Tu J., Carlson M. (1995). REG1 binds to protein phosphatase type 1 and regulates glucose repression in *Saccharomyces cerevisiae*. EMBO J.

[183] Sanz P., Alms G.R., Haystead T.A., Carlson M. (2000). Regulatory interactions between the Reg1-Glc7 protein phosphatase and the Snf1 protein kinase. Mol. Cell Biol.

[184] McCartney R.R., Schmidt M.C. (2001). Regulation of Snf1 kinase. Activation requires phosphorylation of threonine 210 by an upstream kinase as well as a distinct step mediated by the Snf4 subunit. J. Biol. Chem.

[185] Rubenstein E.M., McCartney R.R., Zhang C., Shokat K.M., Shirra M.K., Arndt K.M., Schmidt M.C. (2008). Access denied: Snf1 activation loop phosphorylation is controlled by availability of the phosphorylated threonine 210 to the PP1 phosphatase. J. Biol. Chem.

[186] Hedbacker K., Carlson M. (2006). Regulation of the nucleocytoplasmic distribution of Snf1-Gal83 protein kinase. Eukaryot Cell.

[187] Young E.T., Dombek K.M., Tachibana C., Ideker T. (2003). Multiple pathways are co-regulated by the protein kinase Snf1 and the transcription factors Adr1 and Cat8. J. Biol. Chem.

[188] Tachibana C., Yoo J.Y., Tagne J.B., Kacherovsky N., Lee T.I., Young E.T. (2005). Combined global localization analysis and transcriptome data identify genes that are directly coregulated by Adr1 and Cat8. Mol Cell Biol.

[189] Usaite R., Jewett M.C., Oliveira A.P., Yates J.R., Olsson L., Nielsen J. (2009). Reconstruction of the yeast Snf1 kinase regulatory network reveals its role as a global energy regulator. Mol. Syst. Biol.

[190] Treitel M.A., Carlson M. (1995). Repression by SSN6-TUP1 is directed by MIG1, a repressor/activator protein. Proc. Natl. Acad. Sci. USA.

[191] Lutfiyya L.L., Iyer V.R., DeRisi J., DeVit M.J., Brown P.O., Johnston M. (1998). Characterization of three related glucose repressors and genes they regulate in *Saccharomyces cerevisiae*. Genetics.

[192] Lutfiyya L.L., Johnston M. (1996). Two zinc-finger-containing repressors are responsible for glucose repression of SUC2 expression. Mol. Cell Biol.

[193] Westholm J.O., Nordberg N., Murén E., Ameur A., Komorowski J., Ronne H. (2008). Combinatorial control of gene expression by the three yeast repressors Mig1, Mig2 and Mig3. BMC Genomics.

[194] Tzamarias S., Struhl K. (1995). Distinct TPR motifs of Cyc8 are involved in recruiting the Cyc8-Tup1 corepressor complex to differentially regulated promoters. Genes Dev.

[195] De Vit M.J., Waddle J.A., Johnston M. (1997). Regulated nuclear translocation of the Mig1 glucose repressor. Mol. Biol. Cell.

[196] De Vit M.J., Johnston M. (1999). The nuclear exportin Msn5 is required for nuclear export of the Mig1 glucose repressor of *Saccharomyces cerevisiae*. Curr. Biol.

[197] Rodríguez A., De La Cera T., Herrero P., Moreno F. (2001). The hexokinase 2 protein regulates the expression of the *GLK1*, *HXK1* and *HXK2* genes of *Saccharomyces cerevisiae*. Biochem J.

[198] Young E.T., Kacherovsky N., Van Riper K. (2002). Snf1 protein kinase regulates Adr1 binding to chromatin but not transcription activation. J Biol Chem.

[199] Dombek K.M., Kacherovsky N., Young E.T. (2004). The Reg1-interacting proteins, Bmh1, Bmh2, Ssb1, and Ssb2, have roles in maintaining glucose repression in *Saccharomyces cerevisiae*. J. Biol. Chem.

[200] Hedges D., Proft M., Entian K.D. (1995). CAT8, a new zinc clusterencoding gene necessary for depression of gluconeogenic enzymes in the yeast *Saccharomyces cerevisiae*. Mol. Cell Biol.

[201] Lesage P., Yang X., Carlson M. (1996). Yeast SNF1 protein kinase interacts with SIP4, a C6 zinc cluster transcriptional activator: a new role for SNF1 in the glucose response. Mol. Cell Biol.

[202] Randez-Gil F., Bojunga N., Proft M., Entian K.D. (1997). Glucose derepression of gluconeogenic enzymes in *Saccharomyces cerevisiae* correlates with phosphorylation of the gene activator Cat8p. Mol. Cell Biol.

[203] Vincent O., Carlson M. (1998). Sip4, a Snf1 kinase-dependent transcriptional activator, binds to the carbon source-responsive element of gluconeogenic genes. EMBO J.

[204] Charbon G., Breunig K.D., Wattiez R., Vandenhaute J., Noel-Georis I. (2004). Key role of Ser562/661 in Snf1-dependent regulation of Cat8p in *Saccharomyces cerevisiae* and Kluyveromyces lactis. Mol. Cell Biol.

[205] Vincent O., Carlson M. (1999). Gal83 mediates the interaction of the Snf1 kinase complex with the transcription activator Sip4. EMBO J.

[206] Schmidt M.C., McCartney R.R. (2000). beta-subunits of Snf1 kinase are required for kinase function and substrate definition. EMBO J.

[207] Mayordomo I., Estruch F., Sanz P. (2002). Convergence of the target of rapamycin and the Snf1 protein kinase pathways in the regulation of the subcellular localization of Msn2, a transcriptional activator of STRE (Stress Response Element)-regulated genes. J. Biol. Chem.

[208] De Wever V., Reiter W., Ballarini A., Ammerer G., Brocard C. (2005). A dual role for PP1 in shaping the Msn2-dependent transcriptional response to glucose starvation. EMBO J.

[209] Bertram P.G., Choi J.H., Carvalho J., Chan T.F., Ai W., Zheng X.F. (2002). Convergence of TOR-nitrogen and Snf1-glucose signaling pathways onto Gln3. Mol. Cell Biol.

[210] Imamura K., Ogura T., Kishimoto A., Kaminishi M., Esumi H. (2001). Cell Cycle Regulation via p53 Phosphorylation by a 59-AMP Activated Protein Kinase Activator, 5-Aminoimidazole-4-Carboxamide-1-b-D-Ribofuranoside, in a Human Hepatocellular Carcinoma Cell Line. Biochem and Biophys Res Commun.

[211] Jones R.G., Plas D.R., Kubek S., Buzzai M., Mu J., Xu Y., Birnbaum M.J., Thompson C.B. (2005). AMP-activated protein kinase induces a p53-dependent metabolic checkpoint. Mol. Cell.

[212] Igata M., Motoshima H., Tsuruzoe K., Kojima K., Matsumura T., Kondo T., Taguchi T., Nakamaru K., Yano M., Kukidome D., Matsumoto K., Toyonaga T., Asano T., Nishikawa T., Araki E. (2005). Adenosine monophosphate-activated protein kinase suppresses vascular smooth muscle cell proliferation through the inhibition of cell cycle progression. Circ. Res.

[213] Mandal S., Guptan P., Owusu-Ansah E., Banerjee U. (2005). Mitoch ondrial regulation of cell cycle progression during development as revealed by the tenured mutation in *Drosophila*. Dev. Cell.

[214] Owusu-Ansah E., Yavari A., Mandal S., Banerjee U. (2008). Distinct mitochondrial retrograde signals control the G1-S cell cycle checkpoint. Nat. Genet.

[215] Portillo F., Mulet J.M., Serrano R. (2005). A role for the non-phosphorylated form of yeast Snf1: tolerance to toxic cations and activation of potassium transport. FEBS Lett.

[216] von Plehwe U., Berndt U., Conz C., Chiabudini M., Fitzke E., Sickmann A., Petersen A., Pfeifer D., Rospert S. (2009). The Hsp70 Homolog Ssb is essential for glucose sensing via the SNF1 kinase network. Genes Dev.

[217] Humston E.M., Dombek K.M., Hoggard J.C., Young E.T., Synovec R.E. (2008). Time-dependent profiling of metabolites from Snf1 mutant and wild type yeast cells. Anal. Chem.

[218] Estruch F., Treitel M.A., Yang X., Carlson M (1992). N-terminal mutations modulate yeast SNF1 protein kinase function. Genetics.

[219] Pessina S., Tsiarentsyeva V., Busnelli S., Vanoni M., Alberghina L., Coccetti P. (2010). Snf1/AMPKpromotes S-phase entrance by controlling *CLB5* transcription in budding yeast. Cell Cycle.

[220] Tanaka S., Tak Y.S., Araki H. (2007). The role of CDK in the initiation step of DNA replication in eukaryotes. Cell Div.

[221] Brümmer A., Salazar C., Zinzalla V., Alberghina L., Höfer T. (2010). Mathematical modelling of DNA replication reveals a trade-off between coherence of origin activation and robustness against rereplication. PLoS Comp. Biol.

[222] Liu Y., Xu X., Kuo M.H. (2010). Snf1p regulates gcn5p transcriptional activity by antagonizing spt3p. Genetics.

[223] Huang D., Kaluarachchi S., van Dyk D., Friesen H., Sopko R., Ye W., Bastajian N., Moffat J., Sassi H., Costanzo M., Andrews B.J. (2009). Dual regulation by pairs of cyclin-dependent protein kinases and histone deacetylases controls G1 transcription in budding yeast. PLoS Biol.

[224] Wang H., Carey L.B., Cai Y., Wijnen H., Futcher B. (2009). Recruitment of Cln3 cyclin to promoters controls cell cycle entry via histone deacetylase and other targets. PLoS Biol.

[225] Takahata S., Yu Y., Stillman D.J. (2009). The E2F functional analogue SBF recruits the Rpd3(L) HDAC, via Whi5 and Stb1, and the FACT chromatin reorganizer, to yeast G1 cyclin promoters. EMBO J.

[226] Cantó C., Gerhart-Hines Z., Feige J.N., Lagouge M., Noriega L., Milne J.C., Elliott P.J., Puigserver P., Auwerx J. (2009). AMPK regulates energy expenditure by modulating NAD+ metabolism and SIRT1 activity. Nature.

[227] Gadura N., Michels C.A. (2006). Sequences in the N-terminal cytoplasmic domain of *Saccharomyces cerevisiae* maltose permease are required for vacuolar degradation but not glucose-induced internalization. Curr. Genet.

[228] Gadura N., Robinson L.C., Michels C.A. (2006). Glc7-Reg1 phosphatase signals to Yck1,2 casein kinase 1 to regulate transport activity and glucose-induced inactivation of Saccharomyces maltose permease. Genetics.

[229] Chaves R.S., Herrero P., Moreno F. (1999). Med8, a subunit of the mediator CTD complex of RNA polymerase II, directly binds to regulatory elements of SUC2 and HXK2 genes. Biochem. Biophys. Res. Commun.

[230] Palomino A., Herrero P., Moreno F. (2005). Rgt1, a glucose sensing transcription factor, is required for transcriptional repression of the HXK2 gene in *Saccharomyces cerevisiae*. Biochem. J.

[231] Wanke V., Vavassori M., Thevelein J.M., Tortora P., Vanoni M. (1997). Regulation of maltose utilization in *Saccharomyces cerevisiae* by genes of the RAS/protein kinase A pathway. FEBS Lett.

[232] Vanoni M., Vai M., Popolo L., Alberghina L. (1983). Structural heterogeneity in populations of the budding yeast Saccharomyces cerevisiae. J. Bacteriol.

[233] Lord P.G., Wheals A.E. (1983). Rate of cell cycle initiation of yeast cells when cell size is not a rate-determining factor. J. Cell Sci.

[234] Brauer M.J., Huttenhower C., Airoldi E.M., Rosenstein R., Matese J.C., Gresham D., Boer V.M., Troyanskaya O.G., Botstein D. (2008). Coordination of growth rate, cell cycle, stress response, and metabolic activity in yeast. Mol. Biol. Cell.

[235] Lu C., Brauer M.J., Botstein D. (2009). Slow growth induces heat-shock resistance in normal and respiratory-deficient yeast. Mol. Biol. Cell.

[236] Bradley P.H., Brauer M.J., Rabinowitz J.D., Troyanskaya O.G. (2009). Coordinated concentration changes of transcripts and metabolites in *Saccharomyces cerevisiae*. PLoS Comput. Biol.

[237] Castrillo J.I., Zeef L.A., Hoyle D.C., Zhang N., Hayes A., Gardner D.C., Cornell M.J., Petty J., Hakes L., Wardleworth L., Rash B., Brown M., Dunn W.B., Broadhurst D., O’Donoghue K., Hester S.S., Dunkley T.P., Hart S.R., Swainston N., Li P., Gaskell S.J., Paton N.W., Lilley K.S., Kell D.B., Oliver S.G. (2007). Growth control of the eukaryote cell: a systems biology study in yeast. J Biol.

[238] Levy S., Ihmels J., Carmi M., Weinberger A., Friedlander G., Barkai N. (2007). Strategy of transcription regulation in the budding yeast. PLoS One.

[239] Youk H., van Oudenaarden A. (2009). Growth landscape formed by perception and import of glucose in yeast. Nature.

[240] Bhalla U.S., Iyengar R. (1999). Emergent properties of networks of biological signaling pathways. Science.

[241] Thevelein J.M. (1994). Signal transduction in yeast. Yeast.

[242] Markwardt D.D., Garrett J.M., Eberhardy S., Heideman W. (1995). Activation of the Ras/cyclic AMP pathway in the yeast *Saccharomyces cerevisiae* does not prevent G1arrest in response to nitrogen starvation. J. Bacteriol.

[243] Martin D.E., Hall M.N. (2005). The expanding TOR signaling network. Curr. Opin. Cell Biol.

[244] Wullschleger S., Loewith R., Hall M.N. (2006). TOR signaling in growth and metabolism. Cell.

[245] Martin D.E., Powers T., Hall M.N. (2006). Regulation of ribosome biogenesis: where is TOR?. Cell Metab.

[246] De Virgilio C., Loewith R. (2006). The TOR signalling network from yeast to man. Int. J. Biochem. Cell Biol.

[247] Loewith R., Jacinto E., Wullschleger S., Lorberg A., Crespo J.L., Bonenfant D., Oppliger W., Jenoe P., Hall M.N. (2002). Two TOR complexes, only one of which is rapamycin sensitive, have distinct roles in cell growth control. Mol. Cell.

[248] Wedaman K.P., Reinke A., Anderson S., Yates J., McCaffery J.M., Powers T. (2003). Tor kinases are in distinct membrane-associated protein complexes in *Saccharomyces cerevisiae*. Mol. Biol. Cell.

[249] Düvel K, Santhanam A., Garrett S., Schneper L., Broach J.R. (2003). Multiple roles of Tap42 in mediating rapamycin-induced transcriptional changes in yeast. Mol. Cell.

[250] Dechant R., Peter M. (2008). Nutrient signals driving cell growth. Curr. Opin. Cell Biol.

[251] Inoki K., Guan K.L. (2006). Complexity of the TOR signaling network. Trends Cell Biol.

[252] Barbet N.C., Schneider U., Helliwell S.B., Stansfield I., Tuite M.F., Hall M.N. (1996). TOR controls translation initiation and early G1 progression in yeast. Mol. Biol. Cell.

[253] Zinzalla V., Graziola M., Mastriani A., Vanoni M., Alberghina L. (2007). Rapamycin-mediated G1 arrest involves regulation of the Cdk inhibitor Sic1 in *Saccharomyces cerevisiae*. Mol. Microbiol.

[254] Shamji A.F., Kuruvilla F.G., Schreiber S.L. (2000). Partitioning the transcriptional program induced by rapmycin among the effectors of the Tor proteins. Curr. Biol.

[255] Komeili A., Wedaman K.P., O’Shea E.K., Powers T. (2000). Mechanism of metabolic control. Target of rapamycin signaling links nitrogen quality to the activity of the Rtg1 and Rtg3 transcription factors. J Cell Biol.

[256] Dilova I., Aronova S., Chen J.C., Powers T. (2004). Tor signaling and nutrient-based signals converge on Mks1p phosphorylation to regulate expression of Rtg1.Rtg3p-dependent target genes. J. Biol. Chem.

[257] Tate J.J., Cox K.H., Rai R., Cooper T.G. (2002). Mks1p is required for negative regulation of retrograde gene expression in *Saccharomyces cerevisiae* but does not affect nitrogen catabolite repression-sensitive gene expression. J. Biol. Chem.

[258] Jiang Y., Broach J.R. (1999). Tor proteins and protein phosphatase 2A reciprocally regulate Tap42 in controlling cell growth in yeast. EMBO J.

[259] Düvel K, Broach JR. (2004). The role of phosphatases in TOR signaling in yeast. Curr Top Microbiol Immunol.

[260] Urban J., Soulard A., Huber A., Lippman S., Mukhopadhyay D., Deloche O., Wanke V., Anrather D., Ammerer G., Riezman H., Broach J.R., De Virgilio C., Hall M.N., Loewith R. (2007). Sch9 is a major target of TORC1 in *Saccharomyces cerevisiae*. Mol. Cell.

[261] Mayer C., Grummt I. (2006). Ribosome biogenesis and cell growth: mTOR coordinates transcription by all three classes of nuclear RNA polymerases. Oncogene.

[262] Li H., Tsang C.K., Watkins M., Bertram P.G., Zheng X.F. (2006). Nutrient regulates Tor1 nuclear localization and association with rDNA promoter. Nature.

[263] Aronova S., Wedaman K., Anderson S., Yates J., Powers T. (2007). Probing the membrane environment of the TOR kinases reveals functional interactions between TORC1, actin, and membrane trafficking in *Saccharomyces cerevisiae*. Mol. Biol. Cell.

[264] Sturgill T.W., Cohen A., Diefenbacher M., Trautwein M., Martin D.E., Hall M.N. (2008). TOR1 and TOR2 have distinct locations in live cells. Eukaryot Cell.

[265] Rohde J.R., Bastidas R., Puria R., Cardenas M.E. (2008). Nutritional control via Tor signaling in *Saccharomyces cerevisiae*. Curr Opin. Microbiol.

[266] Lempiäinen H., Uotila A., Urban J., Dohnal I., Ammerer G., Loewith R., Shore D. (2009). Sfp1 interaction with TORC1 and Mrs6 reveals feedback regulation on TOR signaling. Mol. Cell.

[267] Schawalder S.B., Kabani M., Howald I., Choudhury U., Werner M., Shore D. (2004). Growth-regulated recruitment of the essential yeast ribosomal protein gene activator Ifh1. Nature.

[268] Wade J.T., Hall D.B., Struhl K. (2004). The transcription factor Ifh1 is a key regulator of yeast ribosomal protein genes. Nature.

[269] Rudra D., Zhao Y., Warner J.R. (2005). Central role of Ifh1p-Fhl1p interaction in the synthesis of yeast ribosomal proteins. EMBO J.

[270] Mai B., Breeden L. (1997). Xp1, a stress-induced transcriptional repressor of the *Saccharomyces cerevisiae* Swi4/Mbp1 family. Mol. Cell Biol.

[271] Mai B., Breeden L.L. (2006). Identification of target genes of a yeast transcriptional repressor. Methods Mol. Biol.

[272] Ubersax J.A., Woodbury E.L., Quang P.N., Paraz M., Blethrow J.D., Shah. K., Shokat K.M., Morgan D.O. (2003). Targets of the cyclin-dependent kinase Cdk1. Nature.

[273] Jorgensen P., Tyers M. (2004). How cells coordinate growth and division. Curr Biol.

[274] Pringle J.R., Hartwell L.H., Strathern J.N., Jones E.W., Broach J.R. (1981). The *Saccharomyces cerevisiae* cell cycle. The Molecular Biology of the Yeast Saccharomyces cerevisiae: Life Cycle and Inheritance.

[275] Bloom J., Cross F.R. (2007). Multiple levels of cyclin specificity in cell-cycle control. Nat. Rev. Mol. Cell Biol.

[276] Porro D., Vai M., Vanoni M., Alberghino L., Hatzis C. (2009). Analysis and modeling of growing budding yeast populations at the single cell level. Cytometry.

[277] Mitchison J.M. (1971). The Biology of the Cell Cycle.

[278] Hartwell L.H., Unger M.W. (1977). Unequal division in *Saccharomyces cerevisiae* and its implications for the control of cell division. J. Cell Biol.

[279] Johnston G.C., Ehrhardt C.W., Lorincz A., Carter B.L. (1979). Regulation of cell size in the yeast *Saccharomyces cerevisiae*. J. Bacteriol.

[280] Porro D., Brambilla L., Alberghina L. (2003). Glucose metabolism and cell size in continuous cultures of *Saccharomyces cerevisiae*. FEMS Microbiol. Lett.

[281] Alberghina L., Coccetti P., Orlandi I. (2009). Systems biology of the cell cycle of *Saccharomyces cerevisiae*: From network mining to system-level properties. Biotechnol. Adv.

[282] Vanoni M., Rossi R.L., Querin L., Zinzalla V., Alberghina L. (2005). Glucose modulation of cell size in yeast. Biochem. Soc. Trans.

[283] Barberis M., Klipp E., Vanoni M., Alberghina L. (2007). Cell size at S phase initiation: an emergent property of the G1/S network. PLoS Comput. Biol.

[284] Mendenhall M.D., Hodge A.E. (1998). Regulation of Cdc28 cyclin-dependent protein kinase activity during the cell cycle of the yeast *Saccharomyces cerevisiae*. Microbiol. Mol. Biol. Rev.

[285] Rupes I. (2002). Checking cell size in yeast. Trends Genet.

[286] Di T.S., Skotheim J.M., Bean J.M., Siggia E.D., Cross F.R. (2007). The effects of molecular noise and size control on variability in the budding yeast cell cycle. Nature.

[287] Drebot M.A., Barnes C.A., Singer R.A., Johnston G.C. (1990). Genetic assessment of stationary phase for cells of the yeast *Saccharomyces cerevisiae*. J. Bacteriol.

[288] Anghileri P., Branduardi P., Sternieri F., Monti P., Visintin R., Bevilacqua A., Alberghina L., Martegani E., Baroni M.D. (1999). Chromosome separation and exit from mitosis in budding yeast: dependence on growth revealed by cAMP-mediated inhibition. Exp. Cell Res.

[289] Schneper L.A., Krauss R., Miyamoto S., Fang S., Broach J.R. (2004). The Ras/protein kinase A pathway acts in parallel with the Mob2/Cbk1 pathway to effect cell cycle progression and proper bud site selection. Eukaryot. Cell.

[290] Baroni M.D., Martegani E., Monti P., Alberghina L. (1989). Cell size modulation by CDC25 and RAS2 genes in *Saccharomyces cerevisiae*. Mol. Cell. Biol.

[291] Mitsuzawa H. (1994). Increases in cell size at START caused by hyperactivation of the cAMP pathway in *Saccharomyces cerevisiae*. Mol. Gen. Genet.

[292] Baroni M.D., Monti P., Marconi G., Alberghina L. (1992). cAMP-mediated increase in the critical cell size required for the G1 to S transition in *Saccharomyces cerevisiae*. Exp. Cell Res.

[293] Baroni M.D., Monti P., Alberghina L. (1994). Repression of growth-regulated G1 cyclin expression by cyclic AMP in budding yeast. Nature.

[294] Tokiwa G., Tyers M., Volpe T., Futcher B. (1994). Inhibition of G1 cyclin activity by the Ras/cAMP pathway in yeast. Nature.

[295] Belotti F., Tisi R., Martegani E. (2006). The N-terminal region of the *Saccharomyces cerevisiae* RasGEF Cdc25 is required for nutrient dependent cell-size regulation. Microbiology.

[296] Cameron S., Levin L., Zoller M., Wigler M. (1988). cAMP-independent control of sporulation, glycogen metabolism, and heat shock resistance in S. cerevisiae. Cell.

[297] Jorgensen P., Nishikawa J.L., Breitkreutz B.J., Tyers M. (2002). Systematic identification of pathways that couple cell growth and division in yeast. Science.

[298] Levy S, Barkai N. (2009). Coordination of gene expression with growth rate: a feedback or a feed-forward strategy?. FEBS Lett.

